# A Mitogenome-Based Phylogenetic Framework for Mantidae (Mantodea: Mantoidea): Systematic Implications and Spatio-Temporal Dynamics

**DOI:** 10.3390/insects17080830

**Published:** 2026-08-10

**Authors:** Wen-Ning Zhang, Ke Li, Shu-Lin Yao, Jia-Yong Zhang, Yue Ma

**Affiliations:** College of Life Sciences, Zhejiang Normal University, Jinhua 321004, China; 1722759292@zjnu.edu.cn (W.-N.Z.); like20200827@126.com (K.L.); yaoshulin@zjnu.edu.cn (S.-L.Y.); zhang3599533@163.com (J.-Y.Z.)

**Keywords:** Mantidae, mitogenome, phylogeny, divergence time estimation, historical biogeography

## Abstract

Mantidae exhibits the most extensive adaptive radiation within the order Mantodea; however, studies elucidating their internal phylogenetic relationships and spatio-temporal diversification patterns remain scarce. This study aims to resolve the long-standing taxonomic uncertainties within Mantidae by testing the hypothesis that the polyphyletic nature of several subfamilies—specifically Tenoderinae and Hierodulinae—is linked to an incomplete understanding of their mitochondrial genomic evolution. To address this, we assembled 16 complete Mantodea mitochondrial genomes, including seven representatives of the tribe Archimantini (Mantidae: Hierodulinae). Of these, 10 mitogenomes conform to the ancestral insect mitochondrial gene order proposed by Cameron and colleagues, but six mitochondrial gene orders do not conform to the ancestral insect mitochondrial gene order. We identified typical mantodean mitochondrial rearrangement patterns in three species, characterized by gene duplications within the trnA-trnR-trnN-trnS-trnE-trnF and CR-trnI-trnQ-trnM-trnM-ND2 gene cluster. Phylogenetic analyses corroborate the monophyly of Orthoderinae, Mantinae, Choeradodinae, Vatinae, and Archimantini within Mantidae; conversely, Tenoderinae and Hierodulinae are resolved as polyphyletic assemblages. Divergence time estimation indicates that the split between extant Mantodea and Blattodea occurred during the Middle Permian (275.4 Ma, 95% HPD: 207.07–329.09 Ma). Mantidae originated during the Late Cretaceous (87.83 Ma, 95% HPD: 58.89–118.23 Ma), with the diversification of most subfamilies of Mantidae occurring during the Late Cretaceous (80.48 Ma; 95% HPD: 54.86–106.41 Ma). Historical biogeographic analysis and the estimated divergence times suggests that the common ancestor of Mantidae originated within Gondwana during the Late Cretaceous. Ancestral range reconstructions identify Australasia realms and Neotropical realms as the most probable center of origination for Mantidae and achieved a global distribution by expanding along the trans-Antarctic route and leveraging the ‘Indian Ark’ effect.

## Simple Summary

Although Mantidae represents the most extensive adaptive radiation within Mantodea, comprehensive studies on its internal phylogeny and spatio-temporal diversification remain limited. Here, we report the assembly of 16 new mitochondrial genomes, including seven from the tribe Archimantini, to robustly resolve evolutionary relationships within Hierodulinae and Archimantini. We identified characteristic mitochondrial gene rearrangements in six species. Our phylogenetic analyses confirm the monophyly of several Mantidae subfamilies and the tribe Archimantini, while revealing that Tenoderinae and Hierodulinae are polyphyletic. Divergence time estimation indicates that Mantodea and Blattodea diverged in the Middle Permian, with Mantidae originating and undergoing major subfamily diversification in the Late Cretaceous. Biogeographic reconstructions suggest a Gondwanan origin for the Mantidae common ancestor, with Australasia realms and Neotropical realms identified as the primary center of origin. Subsequent global dispersal was facilitated by trans-Antarctic land bridges and the ‘Indian Ark’ hypothesis, closely mirroring the fragmentation history of Gondwana.

## 1. Introduction

Within Dictyoptera, a major superorder of insects, Mantodea represents a highly distinctive and emblematic lineage. Mantises are renowned for their exceptional camouflage strategies and predatory capabilities, and they exhibit modest biodiversity, with approximately 426 described genera and more than 2500 recognized species to date (http://Mantodea.SpeciesFile.org, accessed on 10 July 2026) [1]. As the most species-rich and diverse family in the order Mantodea [2], Mantidae is generally recognized as comprising two primary lineages, the (Choeradodinae + Orthoderinae) clade occupying a basal position. However, the precise phylogenetic placement of Mantinae remains elusive. Multiple previous mitogenomic studies have suggested that Mantinae represents the sister group to the (Choeradodinae + Orthoderinae) clade [3,4,5,6]. In contrast, the phylogenomic analysis by Xu et al. [7], leveraging both transcriptomic and genomic data, recovered a novel topology where Mantinae is grouped with all subfamilies except for the basal (Choeradodinae + Orthoderinae) clade. In addition to these uncertainties, the intricate relationships between Hierodulinae and Tenoderinae, as well as the complex intergeneric relationships within Hierodulinae, remain long-standing unresolved issues. Numerous studies have attempted to resolve the monophyly and internal phylogenetic relationships of Mantidae [5,7]. In 2009, Svenson and Whiting used five mitochondrial genes and four nuclear gene molecular markers and its result showed that the family Mantidae appeared as a paraphyletic group, due to the traditional classification system [8]. In 2019, building upon the 2009 molecular framework, they refine and supplement specific taxa (such as Mantoididae), with the addition of analyses of external morphological characters and male genitalia anatomy [8]. The authors addressed the confounding effects of convergent evolution to propose a more natural classification [2]. The taxonomic scope was expanded to 16 superfamilies, 29 families, 60 subfamilies and 89 tribes. Specifically, Mantidae was divided into 10 subfamilies, 12 tribes and nine subtribes, effectively recovering the monophyly of all familial lineages within the order. Ma et al. [5] demonstrated that *Hierodula* and Tenoderinae are paraphyletic. More recently, Xu et al. [7] successfully recovered Tenoderinae and Hierodulinae as monophyletic lineages. Nevertheless, the generic boundaries of *Hierodula* and *Rhombodera* remain problematic, with both taxa being recovered as polyphyletic assemblages.

As a highly effective molecular genetic marker, the mitochondrial genome has been extensively applied to resolve high-level phylogenetic relationships across a wide range of insect taxa [9,10,11,12]. Despite the continuous accumulation of insect mitochondrial sequences, our understanding of mitochondrial evolution within the order Mantodea remains fragmented. The number of mitogenomes of Mantodea that are available in the NCBI Organelle Genome Resources database (https://www.ncbi.nlm.nih.gov/genome/organelle/, accessed on 10 July 2026) is very high, but still less than 20% of the recorded mantis species [4]. In addition to the primary genomic sequences, mitochondrial gene rearrangements are regarded as highly valuable evolutionary indicators, playing a pivotal role in discriminatory analysis within systematic and taxonomic research [13,14]. Indeed, the mitochondrial architecture of Mantodea is uniquely complex, frequently exhibiting gene rearrangements and large non-coding regions (LNCRs) that contrast with the ancestral insect gene order. These evolutionary events are primarily concentrated in three hotspots: the CR-I-Q-M-ND2, COX2-K-D-ATP8, and ND3-A-R-N-S-E-F-ND5 regions [3,15,16,17,18]. For instance, gene rearrangements within the COX2-K-D-ATP8 cluster are characteristic of Toxoderidae, as exemplified by *Stenotoxodera porioni* and *Toxodera hauseri*, which exhibit varying degrees of K-D gene duplication (where K* and D* denote pseudogenes) [15,16]. Furthermore, tandem duplications of the trnR gene are frequently observed in several lineages, including Hymenopodidae (2–5 copies), the subfamily Mantinae (2–11 copies), and the genus *Theopompa* (2–10 copies) [15,17]. Additionally, extra CR-I-Q-M clusters, often associated with multiple non-coding regions (NCRs), have been widely reported in the family Mantidae [3,15]. While the tandem duplication–random loss (TDRL) model has been widely adopted to explain these rearrangements, many subfamilies, such as Omomantinae and Mellierinae, remain underrepresented in current molecular datasets.

In addition to unresolved phylogenetic affinities, the timing of major divergence events within Mantodea lacks a clear consensus. A substantial discrepancy exists between the fossil record of Mantodea and the divergence times estimated through molecular studies. Indeed, this incongruence is a widespread phenomenon observed across many taxa, such as Hymenoptera and Blattodea [19,20]. While phylogenomic estimations place the origin of Mantodea during the Early Jurassic at approximately 200 Ma [5,8,20,21], the fossil record remains significantly younger, with the earliest known representative, *Jersimantis luzzi,* 1997 [22], appearing only during the Tithonian stage of the Late Jurassic around 149 Ma. Additionally, according to molecular dating by Svenson and Whiting [8], the Mantodea lineage traces back to the Early Jurassic, with the family Mantidae emerging around the Cretaceous–Paleogene (K-Pg) boundary. Legendre et al. [21] posited that the origin of the stem-group Mantodea dates back to approximately 200 Ma, coinciding with the Triassic–Jurassic (T-J) transition. Following this initial emergence, the radiation of the crown-group Mantodea is estimated to have initiated in the Late Jurassic, around 150 Ma. While Evangelista et al. [20] used phylogenomic approaches to date the Dictyoptera origin, placing the Mantodea divergence in the Permian (~263 Ma), their sparse outgroup sampling precluded a definitive estimate for the initial divergence of Mantidae. Using 16 fossil calibrations, Ma et al. [5] dated the crown-group Mantodea to the Late Jurassic and placed the origin of Mantidae in the Late Cretaceous (~88 Ma), with subfamily radiation occurring throughout the Paleogene. In contrast, Xu et al. [7], employing secondary calibrations from prior studies, estimated a significantly younger origin for Mantidae in the Late Paleocene (~65.1 Ma). Collectively, these divergent estimates highlight substantial discrepancies in the inferred divergence times for both Mantodea and Mantidae across different researches.

Plate tectonics profoundly shapes species distribution and biodiversity. Regions of high endemism often coincide with intense tectonic activity, suggesting that the fragmentation of contiguous landmasses accelerated allopatric speciation by impeding gene flow [23]. Furthermore, tectonic movements are frequently coupled with manifestations of climatic heterogeneity. The synergistic driving force of these two factors facilitates the establishment of zoogeographical boundaries [24]. In addition, Southeast Asia and adjacent Australasia represent one of the most geologically dynamic and biologically diverse regions on Earth, shaped by repeated episodes of isolation and re-connection among islands and continental fragments [25,26,27]. Furthermore, previous studies suggested that the phylogenetic structure of Mantodea exhibits a strong geographic signal, rather than being driven by convergent traits or phenotypic resemblance [8,28]. Phylogenetic analyses have shown that traditional family-level classifications within Mantodea are problematic, as the majority of lineages with transcontinental distributions are polyphyletic in origin. In contrast, some clades occurring within the same geographic regions exhibit pronounced divergence in external morphology, yet are shown to be closely related in their evolutionary relationships [8]. This widespread convergence is attributed to the repeated evolution of organisms occupying similar ecological niches, following the fragmentation of Gondwana [8]. Consequently, numerous taxonomic classifications that reflected ecomorphological convergence have been revised to align with geographically-based clades [8], a pattern increasingly observed across other Neoptera lineages [29,30,31].

Within the Mantidae, the phylogenetic placements of numerous subfamilies and tribes remain ambiguous due to a lack of representative sequences, with the tribe Archimantini serving as a prime example of this challenge. In the present study, we sequenced 16 complete mitochondrial genomes from Mantidae and Deroplatyidae, including seven newly obtained sequences from the tribe Archimantini. We reconstructed the phylogenetic tree of Mantodea based on multiple datasets and explored the taxonomic placements of several contentious nodes. Furthermore, we estimated the origin and divergence times of major lineages within the Mantidae and performed historical biogeographic analyses.

## 2. Materials and Methods

### 2.1. Taxon Sampling and DNA Sequencing

In this study, we constructed a comprehensive dataset by integrating 16 newly sequenced complete mitogenomes from Mantidae and Deroplatyidae with publicly available Mantodea mitogenomes and the study of Svenson and Whiting (2009) [8]. Our final taxonomic sampling comprised 91 species representing 4 families, 12 subfamilies and 13 tribes within the order Mantodea (Table S1). In addition, mitochondrial genomes of five Blattodea species (*Blattella germanica*, *Periplaneta americana*, *Cryptocercus relictus*, *Macrotermes barneyi*, and *Mastotermes darwiniensis*) were retrieved from NCBI and designated as outgroup taxa.

Mantises were collected by netting and were stored in 95% alcohol at −20 °C and stored at −80 °C prior to genomic DNA extraction. Total genomic DNA was extracted from the leg muscle tissue using the Ezup Column Animal Genomic DNA Purification Kit (Sangon Biotech, Shanghai, China), in strict accordance with the manufacturer’s standard protocols. Following DNA extraction, the COX1 gene was amplified using the mantid-universal primers TL-J-703 (5′-GGTCAACAAATCATAAAGATATTGG-3′) and TL-N-1311 (5′-TCGAGGTATTCCTGCTAATC-3′). The PCR products were then sent to Zhejiang Youkang Biotechnology Co., Ltd. (Hangzhou, China) for Sanger sequencing. Subsequently, all species were identified based on key diagnostic features of morphology by Dr. Y. Ma and with the NCBI BLAST database (https://blast.ncbi.nlm.nih.gov/Blast.cgi; accessed on 2 June 2025). Detailed information about the samples was included in Table S1. All specimens were assigned a unique code, and corresponding vouchers were stored in Zhang Laboratory, College of Life Sciences, Zhejiang Normal University, Jinhua, China.

Total genomic DNA from the 16 specimens was sequenced using the Next-Generation Sequencing (NGS) platform at BGI Tech Solutions Co., Ltd. (Shenzhen, China). Sequencing was performed on the Illumina HiSeq 2000 platform (BerryGenomics, Beijing, China) with 150 bp paired-end reads. The quality of the raw data was assessed using phyloflash v.3.4.2 [32] and fastp v. 0.23.4 [33]. To enhance the credibility of the final mitogenomes, NOVOPlasty v.4.2 [34], GetOrganelle v.1.7.1 [35] and MitoZ [36] were used to assemble the mitogenomes on the basis of the clean data. After we obtained the same mitochondrial genomes by at least three methods, we judged that the mitogenomes were correct. Subsequently, the clean data were used to assemble the mitogenomes, which were then annotated using MITOS2 on the Galaxy platform (https://usegalaxy.eu; accessed on 31 October 2025) [37]. *tRNA*Scan-SE (http://lowelab.ucsc.edu/tRNAscan-SE/index.htm, accessed on 24 December 2025) [38] and MITOS [37] were used to predict *tRNA* secondary structures. MEGA 11 [39] was employed to verify the accurate translation of protein-coding genes (PCGs) into amino acid sequences. The complete mitogenomes were visualized using Proksee (https://proksee.ca; accessed on 14 November 2025) [40]. All 16 mitochondrial genomes have been deposited in the National Center for Biotechnology Information (NCBI) database. The GenBank accession numbers for these sequences are listed in Table S2.

### 2.2. Dataset Selection

In this study, 15 target genes were extracted from mitochondrial genome files in GenBank format, including 13 protein-coding genes (ATP6, ATP8, COX1–3, CYTB, ND1–6, and ND4L) and 2 ribosomal RNA genes (12S rRNA and 16S rRNA). The extraction was implemented via a custom Python script (1_4.py). This script leverages Biopython 1.83 [41] to parse sequence features, identifies target genes using regular expressions, and exports a separate FASTA file for each gene. The gene extraction information for all sequences is shown in Table S3. Subsequently, multiple sequence alignment was performed for each gene with MAFFT v7.520 [10] (parameters: --auto --thread 8 --adjustdirectionaccurately). Ambiguous regions within the alignments were trimmed using Gblocks v0.91b [11] (parameters: -t=d -b5=h) to retain only conserved segments. Finally, another custom Python script (3.py) concatenated all trimmed genes into a supermatrix following species order, and simultaneously generated a partition file for subsequent phylogenetic tree reconstruction. All analytical procedures were run in a Python 3.10 environment managed by Conda.

### 2.3. Phylogenetic Reconstruction

Phylogenetic relationships were inferred using both Maximum Likelihood (ML) and Bayesian Inference (BI) analyses. For the concatenated datasets of PCGs and rRNAs, the optimal partitioning schemes and best-fit substitution models for each partition were determined using PartitionFinder v2.2.1 [12], employing a gene-based partitioning strategy. Maximum Likelihood (ML) trees were constructed using RAxML v8.2.0 [13] with 1000 bootstrap replicates. Bayesian Inference (BI) trees were reconstructed using MrBayes v3.2.7a [14], with the Markov Chain Monte Carlo (MCMC) chains running for 10 million generations to ensure that the average standard deviation of split frequencies (ASDSF) fell below 0.01. The sampling frequency was set to 1000 generations, and the convergence of the MCMC chains was inspected using Tracer v1.7 [42]. The initial 25% of the sampled trees were discarded as burn-in Tree visualization and aesthetic editing were performed using FigTree v1.4.4. [43] and chiplot (https://www.chiplot.online, accessed on 31 November 2025) [44].

### 2.4. Divergence Time Estimation

To calibrate the phylogenetic tree of Mantodea, 5 fossil calibration points were incorporated (Table 1), primarily sourced from the Fossil Calibration Database (https://paleobiodb.org/classic/displayDownloadGenerator, accessed 14 November 2025), Timetree (http://www.timetree.org/, accessed 24 November 2025) and previous studies [45,46,47,48,49,50]. Divergence times were estimated using the MCMCTree [51] program within the PAML v4.8 package. The topology derived from the Bayesian Inference (BI) analysis was employed as the input tree for the molecular clock dating. The root age was calibrated at 311.0 Ma based on the fossil *Qilianiblatta namurensis* [52] The parameters for MCMCTree were configured as follows: a burn-in period of 400,000 iterations, a sampling frequency of 10, and a total collection of 100,000 samples. Convergence diagnostics were performed using Tracer v1.6. The MCMC runs were considered to have achieved sufficient convergence when the Effective Sample Size (ESS) for all parameters exceeded 200. The final chronogram was visualized and annotated using FigTree v1.4.4 [43] and chiplot (https://www.chiplot.online, accessed on 31 November 2025) [44].

### 2.5. Historical Biogeographic Analysis

The pruning process was performed using ‘drop.tip’ function in the R package ‘ape’ [53] to retain only taxa belonging to the family Mantidae. The biogeographical regions are mainly divided based on the zoogeographical realms proposed by Wallace [54], namely the Palearctic, Nearctic, Neotropical, Afrotropical, Indomalaya, and Australasia realms. Additionally, we consider the Indian Peninsula (PIP) and Madagascar as separate partitions to reflect the distribution pattern of Mantidae, as they were once distinct fragments of the Gondwana landmass. In total, 8 areas were defined: Palearctic (Europe, Africa and Arabia north of the Tropic of Cancer, and Asia north of the Himalayas and the Qinling Mountains), Nearctic (Most of North America, Greenland and Northern Mexico), Neotropical (Carribean islands and Central/South America), Afrotropical (Sub-Saharan Africa including), PIP (present-day Indian Peninsula along with Sri Lanka, parts of Pakistan and Bangladesh), SE Asia (continental SE Asia along with western Malesia that was part of Gondwana; referred to as the Cimmerian terrane), Madagascar, and Australasia realms (Australia, New Zealand, New Guinea, New Britain, New Ireland, and surrounding islands). The geographical distribution data of species (Table S4) were mainly derived from our observation and sampling records, referred to original literature published online and supplemented with iNaturalist website (https://inaturalist.ala.org.au/, accessed on 30 May 2025). The global biogeographic history of Mantidae was assessed with BioGeoBEARS [55,56] implemented in RASP 4.2 [57]. According to the model test results (Table S5), We employed the DEC model [58] to do this analysis.

## 3. Results

### 3.1. Composition and Organization of Mitogenomes

In this study, a total of 16 complete mitogenomes from the families Mantidae and Deroplatyidae were obtained. The genome length ranged from 15,427 bp in *Oxyopsis peruviana* to 17,049 bp in *Archimantis latistyla* (Figure S1). Of these, 10 mitogenomes contained the standard set of 37 genes, including 13 protein-coding genes (PCGs), 22 transfer RNA genes (*tRNA*s) (Figure S2), and two ribosomal RNA genes (rRNAs), along with a non-coding control region (CR). They conform to the ancestral insect mitochondrial gene order proposed by Cameron and colleagues. In addition, six mitochondrial gene orders do not conform to the ancestral insect mitochondrial gene order, namely those of *Rhombodera basalis*, *Oxyopsis peruviana*, *Hierodula timorensis*, *Euchomenella* sp., *Orthodera novaezealandiae*, and *Phasmomantella* sp. To avoid misjudgments caused by sequencing issues, we designed specific primers (Table S6) to verify gene rearrangements.

First, a duplication of the trnR gene was observed within the ND3-trnR-trnN-trnS*1*-trnE-trnF-ND5 gene cluster in *Euchomenella* sp., *Orthodera novaezealandiae*, and *Phasmomantella* sp. Both copies of the trnR gene were identical in sequence to each other, and both retained the ancestral anticodon TCG. This sequence identity suggests a relatively recent duplication event or the presence of concerted evolution maintaining the homogeneity of the two gene copies. In *Orthodera novaezealandiae*, a 17 bp intergenic spacer (IGS) was identified between the two trnR copies. Notably, a 12 bp segment within this spacer was found to be identical to the 3′ end of the upstream trnA, suggesting that this spacer represents a degraded residue of a previously duplicated trnA gene. Second, there is a gene rearrangement in the CR-trnI-trnQ-trnM-ND2 gene cluster in *Rhombodera basalis*, *Oxyopsis peruviana*, *Hierodula timorensis*. *Rhombodera basalis* and *Hierodula timorensis* are CR-trnI-trnQ-trnM-trnI-trnQ-trnM-trnM-ND2 and *Oxyopsis peruviana* is CR-trnI-trnQ-trnM-trnM-ND2. In *Rhombodera basalis* and *Hierodula timorensis*, the second trnI, trnQ and trnM are same with the first trnI, trnQ and trnM. As for the last trnM, however, possibly due to random loss and transversion, several differences were found compared to the original genes.

### 3.2. Phylogenetic Analyses

**Figure 1:**
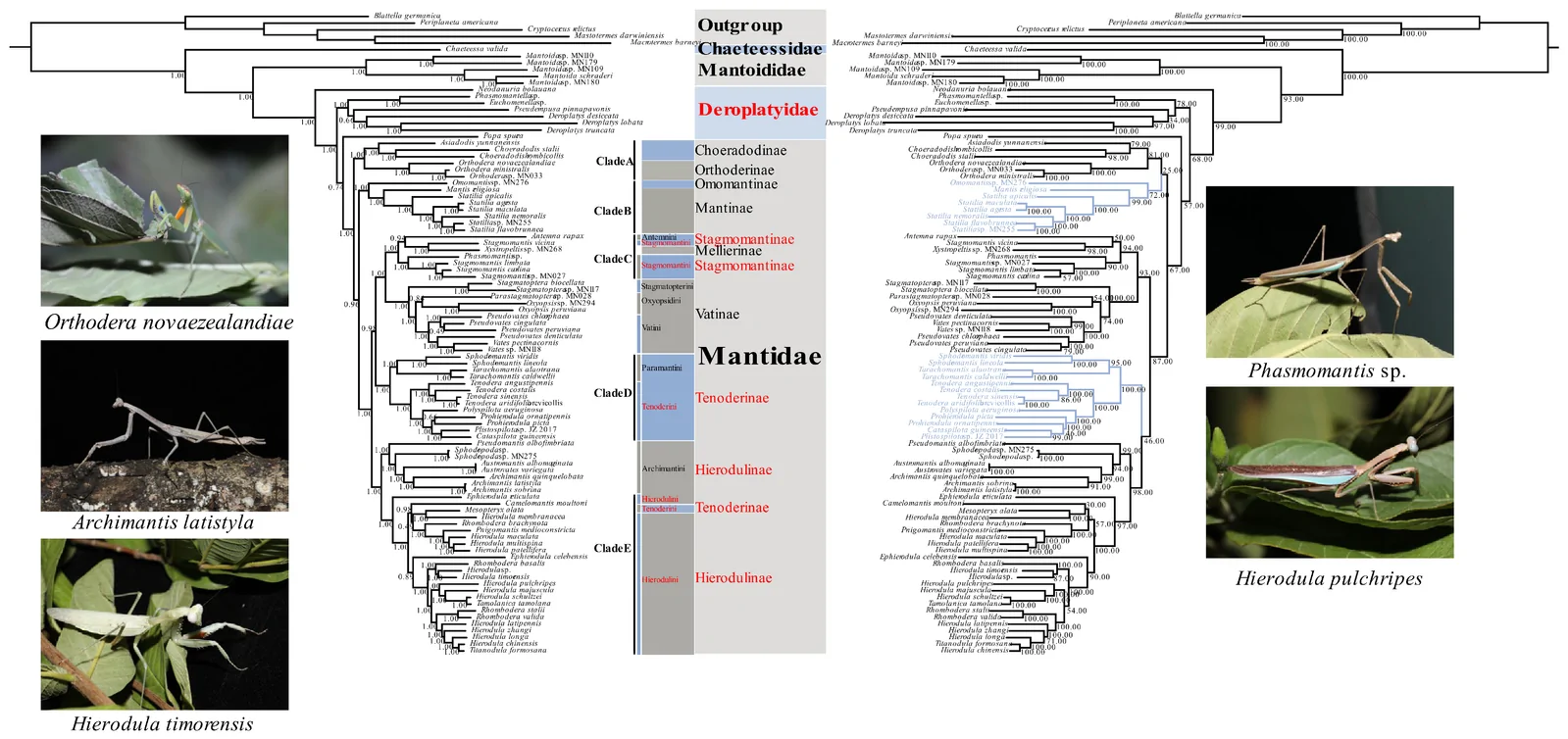
Bayesian phylogeny (**left**) and Maximum Likelihood phylogeny (**right**) of Mantodea inferred from the CDS + RNA dataset. Bootstrap values and posterior probabilities are shown on the branches. The branches marked in blue on the ML tree on the right side of the figure represent the incongruent branches between the ML-tree and BI-tree results. The families, subfamilies and tribes marked in red indicate non-monophyletic groups using the Schwarz and Roy [2] classification system. The ecological photos are from this study.

The ML and BI analyses (Figure 1) yielded topological structures with certain discrepancies. The differences between BI tree and ML tree lies in the phylogenetic position of Mantinae, Omomantinae and Tenoderinae. In the ML tree, Mantinae and Omomantinae form the sister group to the clade comprising Choeradodinae and Orthoderinae (BS: 25). Tenoderinae is the sister group to Hierodulinae (BS: 46). In the BI tree, the clade containing Mantinae and Omomantinae is the sister group to all other Mantidae subfamilies except for Choeradodinae and Orthoderinae (PP: 0.96). Tenoderinae occupies the sister position to the clade comprising Vatinae, Stagmomantinae, and Mellierinae (PP: 0.98). Because the BI tree provided higher nodal support, it was selected as the reference topology for all subsequent divergence time estimations and ancestral area reconstructions.

Both ML and BI analyses consistently recovered Chaeteessidae as the basal-most lineage of extant Mantodea, and it forms a sister group to all remaining Mantodea families. The monophyly of the family Mantidae was strongly supported (PP: 1.00, BS: 67). At the subfamily level, the monophyly of Choeradodinae (PP: 1.00, BS: 79), Orthoderinae (PP: 1.00, BS: 100), Mantinae (PP: 1.00, BS: 99), and Vatinae (PP: 1.00, BS: 100) within Mantidae is well supported. In contrast, the placement of *Mesopteryx alata* within Hierodulinae renders both Tenoderinae and Hierodulinae non-monophyletic. After removing the sequence of *Mesopteryx alata*, Tenoderinae and Hierodulinae can recover their monophyly. Our Bayesian phylogenetic analyses resolved a robust topology for this group, recovering Choeradodinae and Orthoderinae as sister taxa that collectively constitute the basal clade of Mantidae.

Furthermore, our phylogenetic analysis recovered the monophyly of five tribes: Stagmatopterini (PP: 1.00, BS: 100), Oxyopsidini (PP: 0.84, BS: 54), Vatini (PP: 1.00, BS: 100), Paramantini (PP: 1.00, BS: 95), and Archimantini (PP: 1.00, BS: 99). At the subtribe level,, Paramantina (PP: 1.00, BS: 100), Tarachomantina (PP: 1.00, BS: 100), Polyspilotina (PP: 1.00, BS: 100), Trachymantina (PP: 1.00, BS: 100), and Archimantina (PP: 1.00, BS: 99) are monophyletic. Within Archimantini, our phylogenetic reconstruction recovered (Pseudomantina + (Trachymantina + Archimantina)). Within Archimantina, the monophyly of *Archimantis* (PP: 1.00, BS: 91) and *Sphodropoda* (PP: 1.00, BS: 100) received strong support. The internal topology of Archimantina is defined as *Archimantis* + (*Austromantis* + *Austrovates*).

### 3.3. Divergence Time Estimation

**Figure 2:**
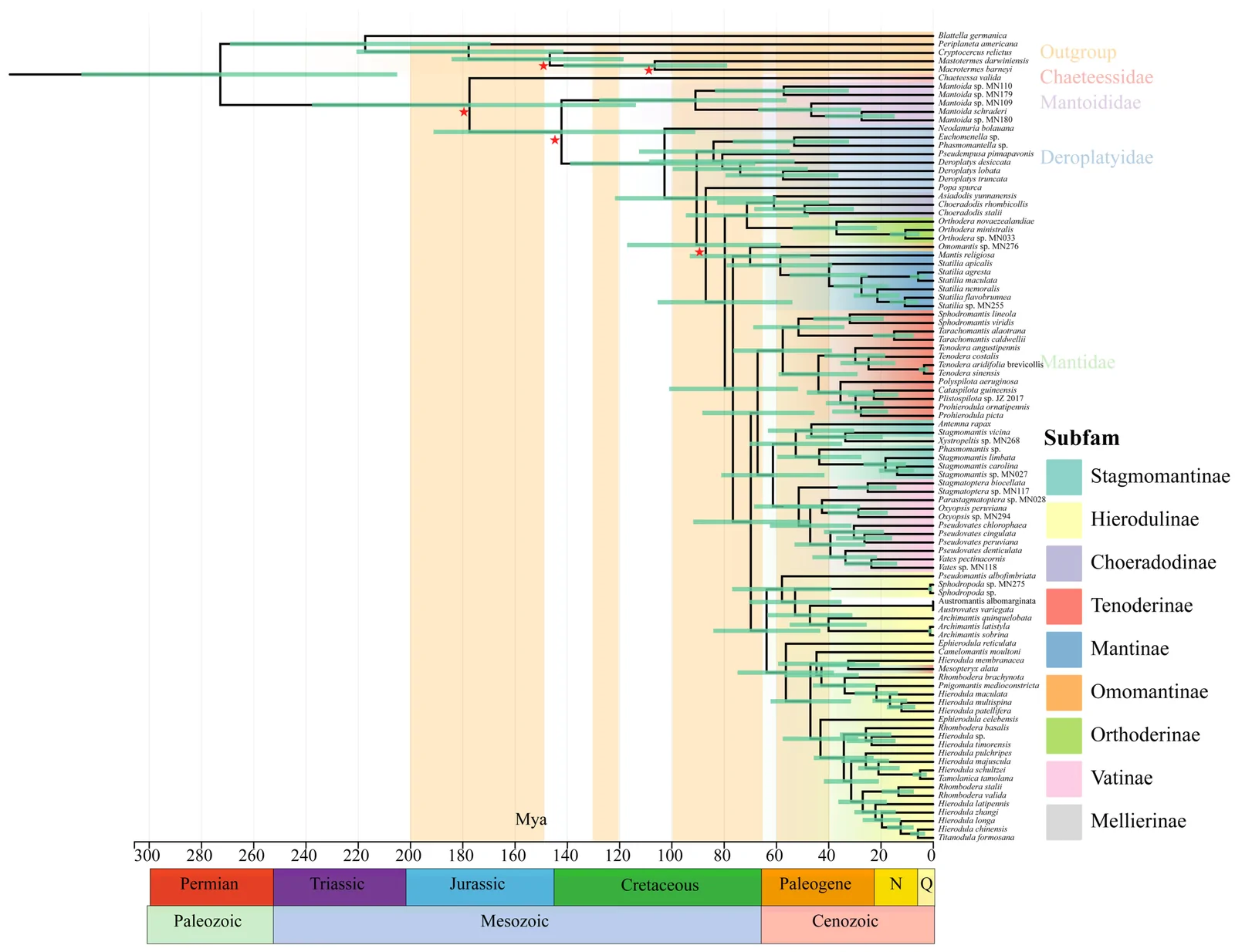
The chronogram of Mantodea. The divergence time estimation of Mantodea was inferred using MCMCTree, with the 95% highest posterior density (HPD) intervals indicated by bars. Stars on the nodes denote the placement of the fossil calibrations. The timeline at the bottom is in millions of years. Q is the abbreviation for Quaternary, and N is the abbreviation for Neogene. Pale yellow bars behind phylogeny indicate the following events: split between East and West Gondwana (200–150 Ma), split between Peninsular Indian Plate and East Gondwana (130–120 Ma), the age of the angiosperm Radiation (100–66 Ma) and collision between Peninsular Indian and Eurasian plates (60–40 Ma).

Based on the BI tree topology, we performed the divergence time estimation. The results (Figure 2) indicate that the divergence between Mantodea and Blattodea occurred during the Late Permian (275.4 Ma, 95% HPD: 207.07–329.09 Ma). As the earliest diverging lineage of the crown-group Mantodea, Chaeteessidae originated during the Early Jurassic, approximately 179.1 Ma (95% HPD: 114.82–239.94 Ma) and Spinomantodea originated during the Early Cretaceous, approximately 143.58 Ma (95% HPD: 91.87–193.01 Ma).

Additionally, Mantidae originated during the Late Cretaceous, approximately 87.83 Ma (95% HPD: 58.89–118.23 Ma). Within Mantidae, the diversification of most subfamilies originated during the Late Cretaceous at 80.48 Ma (95% HPD: 54.36–106.41 Ma). The diverging between Choeradodinae and Orthoderinae was dated to the Late Cretaceous at 71.94 Ma (95% HPD: 48.02–95.49 Ma). Mantinae and Omomantinae originated during the Late Cretaceous, approximately 70.72 Ma (95% HPD: 47.49–93.97 Ma). Tenoderinae originated during the Late Cretaceous, approximately 68.3 Ma (95% HPD: 46.28–89.6 Ma). Subsequently, the split between Vatinae and Stagmomantinae occurred during the Late Cretaceous at 67.82 Ma (95% HPD: 45.87–89.14 Ma). Mellierinae originated during the Late Eocene, approximately 33.96 Ma (95% HPD: 19.45–49.27 Ma). Additionally, the subfamily Hierodulinae originated during the Late Cretaceous at 70.44 Ma (95% HPD: 47.7–92.67 Ma).

Within Hierodulinae, the origin of the tribe Archimantini can be traced back to the early Paleogene at 64.33 Ma (95% HPD: 43.58–84.92 Ma). Within the Archimantini, Pseudomantina is the most ancestral lineage and it originated at 58.39 Ma (95% HPD: 39.29–77.62 Ma). This was followed by the divergence of Trachymantina and Archimantina at 53.32 Ma (95% HPD: 35.41–71.05 Ma). Within Archimantina, *Archimantis* originated at 47.58 Ma (95% HPD: 31.19–64.1 Ma). In contrast, the divergence between *Austromantis* and *Austrovates* occurred much later, commencing at only 0.02 Ma (95% HPD: 0–0.06 Ma).

### 3.4. Historical Biogeographic Analysis

**Figure 3:**
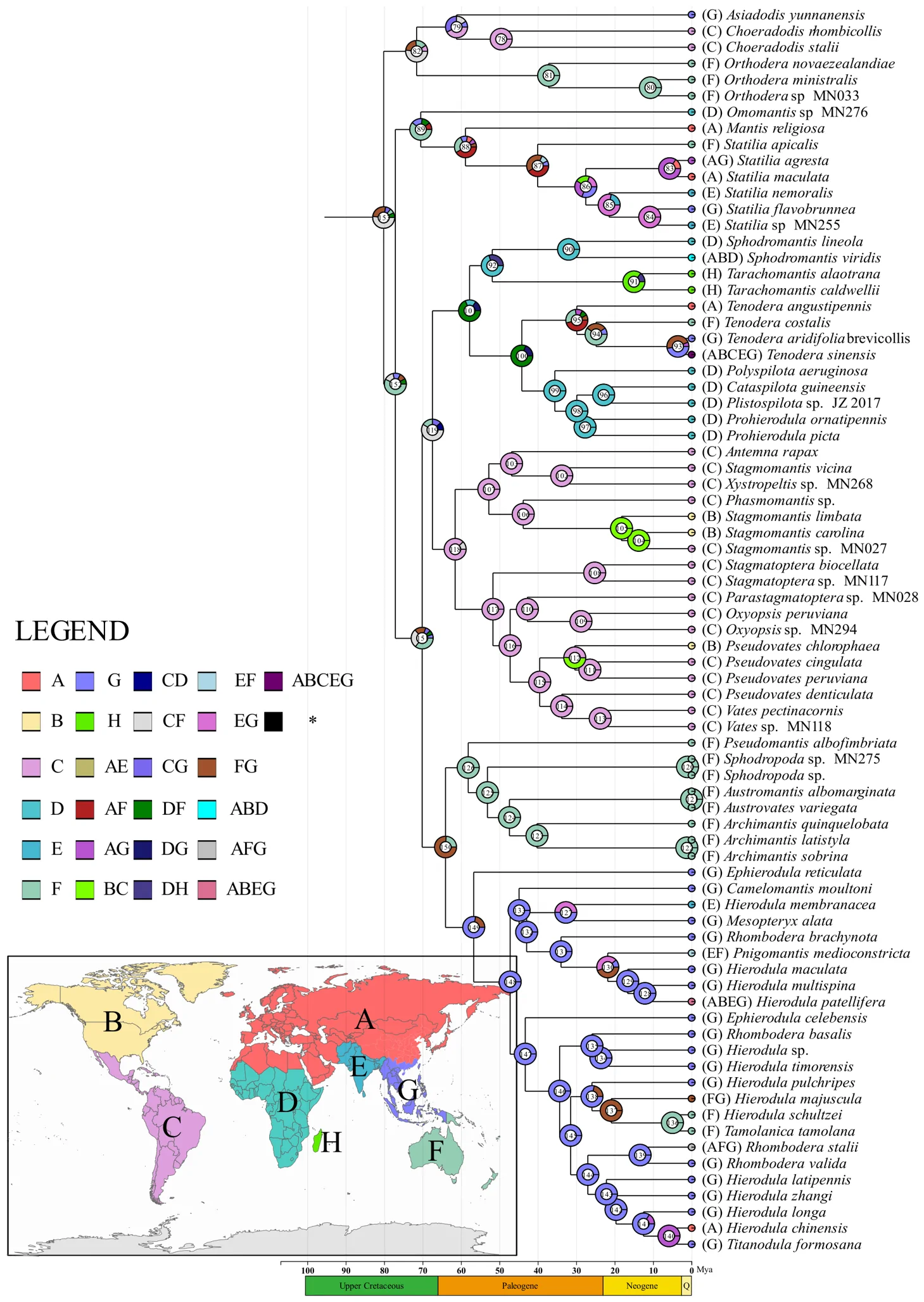
Ancestral range estimation for dated phylogeny of Mantidae. Colored boxes at nodes and tips represent areas as indicated in the inset map (A: Palearctic, B: Nearctic, C: Neotropical, D: Afrotropical, E: PIP, F: Australasia, G: SE Asia, H: Madagascar). The asterisk in legned indicates that the distribution position of this node is unknown.

We mapped the biogeographic regions (Table S4) onto the phylogeny of Mantidae, and it revealed a distinct lineage-specific distribution pattern. As illustrated in Figure 3, we found the extensive phylogenetic and the intermingling of taxa from the PIP, Afrotropical, SE Asia, Madagascar, Australasia and Neotropical realms, which collectively reveals a history of multiple complex intercontinental dispersal events. Our ancestral area reconstruction suggests a 51.74% probability that Mantidae originated in Australasia realms and Neotropical realms at 80.48 Ma. Additionally, Mantidae exhibits a 26.21% probability of originating within the combined regions of the Australasian realm and Southeast Asia. Following its origin, the family diverged into two primary lineages, which subsequently expanded into the Palearctic realms, Nearctic realms and PIP realms. At 71.94 Ma, the ancestor of Choeradodinae and Orthoderinae (node 82) shows a 51.74% probability of distribution within the combined Australasian and Neotropical realms, and a 22.27% probability of distribution within the combined Australasian realm and Southeast Asia. Subsequently, the ancestor of Choeradodinae (node 79) dispersed into Southeast Asia and the Neotropical realm, with a 42.11% probability of distribution in the Neotropical realm and a 32.76% probability of distribution across the combined Neotropical and Southeast Asian realms. In contrast, the ancestor of Orthoderinae (node 81) remained exclusively restricted to the Australasian realm, showing a 100% probability of distribution throughout its evolutionary history. The ancestral distribution of the remaining Mantidae (node 152) shows multiple possibilities, with a 57.30% probability of distribution in the Australasian realm, a 14.36% probability of distribution in the combined Australasian and Neotropical realms, and a 10.80% probability of distribution in Southeast Asia. At 70.72 Ma, the ancestor of Mantinae (node 89) retained a 75.93% probability of occupying the Australasian realm, subsequently dispersing toward the Palearctic and Southeast Asian realms during the Eocene. Simultaneously, the lineage leading to the ancestor of Omomantinae dispersed into the Afrotropical realm. At 67.82 Ma, the ancestor of the clade containing Tenoderinae, Vatinae, Stagmomantinae, and Mellierinae (node 119) shows a 58.82% probability of distribution in the combined Australasian and Neotropical realms, a 16.46% probability in the Australasian realm, and a 13.32% probability in the combined Neotropical and Southeast Asian realms. At 58.11 Ma, the ancestor of Tenoderinae (node 101) dispersed into the Afrotropical and Australasian realms with a 68.70% probability. This lineage subsequently diverged into two branches; the ancestor of the tribe Paramantini (node 92) shows a 70.06% probability of dispersal into the Afrotropical realm, along with a 29.94% probability of dispersal into the combined Afrotropical and Madagascan regions. Within this tribe, the subtribe Paramantina (node 90) has remained restricted to the Afrotropical realm to the present day, whereas the subtribe Tarachomantina (node 91) successfully dispersed into Madagascar. At 44.31 Ma, the ancestor of the tribe Tenoderini (node 100) showed a high probability (80.81%) of remaining in the combined Afrotropical and Australasian realms, before diverging into two lineages. The subtribe Tenoderina (node 95) shows a 45.76% probability of dispersal into the combined Palearctic and Australasian realms, a 27.10% probability of remaining in the Australasian realm, and a 10.67% probability of dispersal into the combined Palearctic and Southeast Asian realms; meanwhile, the subtribe Polyspilotina (node 99) has remained exclusively in the Afrotropical realm (100%) to the present day. At 61.88 Ma, the ancestor of the clade comprising Vatinae, Stagmomantinae, and Mellierinae (node 118) showed an 87.93% probability of remaining within the Neotropical realm. Vatinae, Stagmomantinae, and Mellierinae persisted and diversified within the Neotropical realm until 30.61 Ma, when Pseudovates chlorophaea (within tribe Vatini, node 112) began to disperse into the Nearctic realm, followed by the dispersal of Stagmomantis (node 105) into the Nearctic realm at 18.4 Ma. At 64.93 Ma, the ancestor of Hierodulinae (node 150) shows a 24.07% probability of remaining in the Australasian realm and a 75.93% probability of dispersal into the combined Southeast Asian and Australasian regions. The tribe Archimantini (node 126) maintained its ancestral distribution in the Australasian realm, while the tribe Hierodulini (node 149) began to disperse into Southeast Asia (77.68%).

## 4. Discussion

### 4.1. Mitochondrial Gene Rearrangement Events

**Figure 4:**
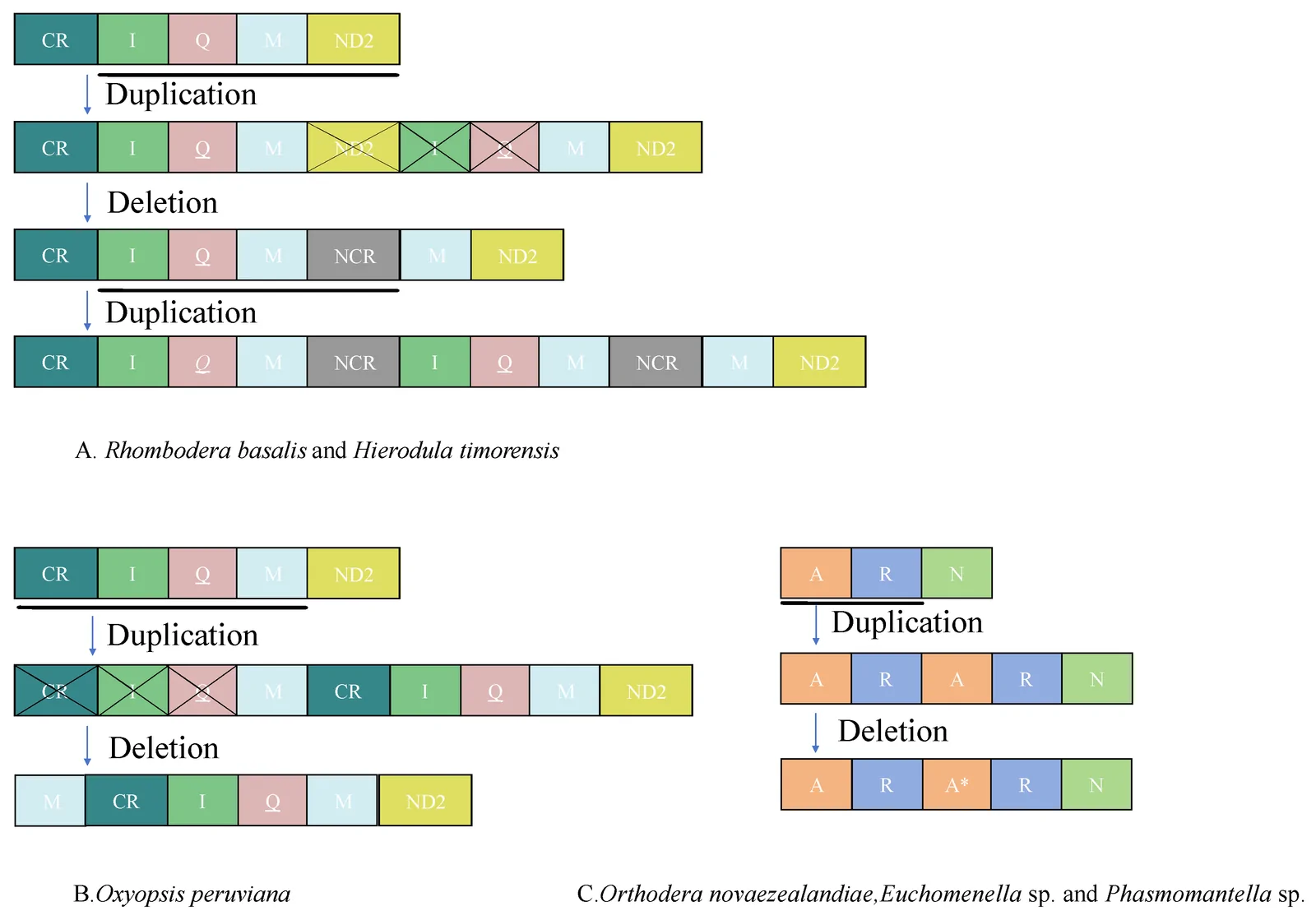
Six mantis mitogenomes with putative mechanism of gene rearrangements. The size of the genes is not proportional. Genes with underlines encoded by the N-strand and those without underlines are genes encoded by the J-strand. Standard abbreviations are used for PCGs, rRNAs, and control regions. The tRNAs are represented by single letters. Asterisked genes indicate pseudogenes. The remaining genes and gene orders are excluded that were identical to those of the ancestral insect. Horizontallines, asterisk symbols, and crossed-out colored boxes represent gene duplications, gene pseudogenization, and gene deletions, respectively.

Mitochondrial gene rearrangement is widely recognized as a pivotal driver of evolutionary processes. Previous studies have highlighted that while the mitochondrial genomes of the vast majority of insects exhibit high structural conservation, such rearrangements are notably frequent within the order Mantodea. The novel gene rearrangements identified within Mantodea are primarily attributed to the duplication, inversion, and translocation of *tRNA* genes, with duplication representing the most prevalent mechanism [4,59]. It is particularly prevalent that duplication events involve trnR within the ND3-trnA-trnR-trnN-trnS-trnE-trnF-ND5 cluster. Gene order rearrangements (GORs) within this specific region have been extensively utilized as informative markers for phylogenetic reconstruction [3,15,17,60,61,62,63]. Previous studies have observed gene duplications across multiple evolutionary lineages within Mantodea, including the superfamilies Gonypetoidea, Haanioidea, Eremiaphilioidea, Hymenopoidea, and Mantoidea, as well as the family Mantidae [4,17,18,64,65]. In summary, Mantodea serves as an ideal model group for advancing our understanding of the mechanisms and evolutionary significance of mitochondrial gene rearrangements. In our newly sequenced mitogenomes, the identified gene rearrangement region falls within the trnA-trnR-trnN cluster, which is a hotspot frequently reported in previous studies of this order. Currently, the tandem duplication–random loss (TDRL) model is widely accepted as the prevailing mechanism for explaining mitochondrial gene rearrangements in insects, and it has been extensively applied in studies concerning the order Mantodea [3,66,67,68]. Therefore, the model is equally applicable to this study (Figure 4).

First, in the CR-trnI-trnQ-trnM-ND2 region, as for *Rhombodera basalis* and *Hierodula timorensis*, the I-Q-M-ND2 cluster was tandem repeated once. Then, the deletion or random loss of the first ND2 and the second trnQ and trnI happened. Then, a tandem repeat of I -Q-M-NCR happened (Figure 4A). This rearrangement mechanism was similar to the description by Xu et al. [3] and Lin et al. [4]. The possible explanation for *Oxyopsis peruviana* was consistent with Zhang and Ye [63] and Lin et al. [4] (Figure 4B).

Second, the mitogenomic gene order of *Orthodera novaezealandiae* is characterized as ND3-trnA-trnR-trnR-trnN-trnS-trnE-trnF-ND5. Given the high sequence similarity between the intergenic region of the two trnR genes and the trnA gene, we hypothesize the following rearrangement pathway: a tandem duplication of the A-R cluster resulted in an ancestral trnA-trnR-trnA-trnR intermediate, followed by the partial deletion of the second trnA copy. The presence of a vestigial sequence between the duplicated trnR genes, which retains significant homology to trnA, provides robust empirical evidence for the TDRL model. This suggests that the rearrangement was not a sudden translocation but a result of slipped-strand mispairing during mtDNA replication. The previously published mitogenome of *Orthodera ministralis* also observed a triple duplication of trnR within this region. The intergenic spacer (IGS) between the first and second trnR copies is identical in sequence and exhibits high homology with trnA. Furthermore, a 91 bp IGS located between the third trnR and trnN shows significant similarity to trnR. Based on these observations, we propose a tandem duplication–random loss (TDRL) pathway: an initial quadruplication of the trnA-trnR cluster resulted in a trnA-trnR-trnA-trnR-trnA-trnR-trnA-trnR intermediate, followed by the random loss of the second, third, and fourth trnA copies, as well as the fourth trnR gene. Consequently, the mitogenome of *Orthodera ministralis* retains three functional trnR genes, along with one vestigial trnR remnant and three degraded trnA residues (Figure 4C). Both species belong to the same genus, but they possess a different number of trnR copies. This indicates that trnR copy number exhibits intergeneric variation. The mitogenomic sequences of *Euchomenella* sp. and *Phasmomantella* sp. can also be explained by the tandem duplication–random loss (TDRL) model. However, given that the mitogenomic gene arrangements in the majority of the aforementioned lineages still conform to the ancestral state, we infer that the origin and evolution of multiple duplications are likely independent events.

### 4.2. Mantidaen Phylogeny

At the subfamily level, Mantidae is divided into two major clades. The clade A is recovered as (Choeradodinae + Orthoderinae). Our analytical results regarding the phylogenetic positions of Orthoderinae and Choeradodinae are consistent with those reported in previous studies [3,4,5,7,8,69]. In our study, the phylogenetic placement of Mantinae is consistent with a transcriptome-based study by Xu et al. [7]. However, it is different from multiple recent studies based on large-scale genomic data [3,4,5,6]. They showed that Mantinae is the sister group to a clade of (Choeradodinae + Orthoderinae). This discrepancy in the phylogenetic position of Mantinae may stem from differences in dataset selection. Given the current controversy, further investigations utilizing more comprehensive genomic datasets and expanded taxon sampling strategies are required to resolve these relationships.

The second clade is recovered as (clade B+ (((clade C+ Vatinae)+ clade D) + (Archimantini+ clade E))). In this topology, it does not support the monophyly of the system proposed by Schwarz and Roy, because Stagmomantinae, Tenoderinae and Hierodulinae are polyphyletic groups. Notably, both the present study and numerous molecular phylogenetic analyses [3,4,5,8,18,21,69,70] consistently recover a close phylogenetic relationship among the genera *Mesopteryx*, *Hierodula*, *Tamolanica*, *Rhombodera*, *Ephierodula*, *Camelomantis*, *Titanodula*, and *Pnigomantis*. Consistent with the aforementioned findings, the genera *Mesopteryx*, *Camelomantis*, *Tamolanica*, *Rhombodera*, *Ephierodula*, *Titanodula* and *Pnigomantis* are nested within a clade containing multiple *Hierodula* species, so it makes the genus *Hierodula* non-monophyletic. The complex and close evolutionary relationships among these genera are further substantiated at the molecular level. Xu et al. [3] identified a prominent mitochondrial synapomorphy: a large non-coding sequence situated in the intergenic spacer between the trnM and ND2 genes. This region, spanning 281 to 1SS541 bp, exhibits high evolutionary conservation in its primary structure, so it can serve as a shared mitochondrial genetic signature for these taxa. *Mesopteryx* was traditionally assigned to Tenoderinae, but it is recovered as nested within the Hierodulinae in our study. This topological arrangement makes both Tenoderinae and Hierodulinae non-monophyletic. This finding deviates from the classical morphological classification system [2], but it is highly congruent with several recent mitogenomic phylogenies [3,4,5,6], all of which support an intimate phylogenetic relationship between *Mesopteryx* and the various genera of Hierodulinae. However, given the unexpected placement of *Mesopteryx* within the tree, we cannot entirely dismiss the potential influence of specimen misidentification or data contamination on our phylogenetic inferences. Regarding the phylogenetic placement of the genus *Mesopteryx*, further in-depth investigations utilizing more representative taxon sampling and multi-omic datasets are essential to resolve its evolutionary history.

Within Hierodulinae, the present study recovered the monophyly of Archimantini and yielded robust support for the topology of (Archimantini + Hierodulini). These findings are congruent with long-standing results from previous phylogenetic research [8].

### 4.3. Divergence Time Estimation

The origin and diversification of Mantidae was dated to the Late Cretaceous period (87.83 Ma), which is generally consistent with the timing observed in previous studies [5,7,8]. Additionally, this time frame coincides with the rapid radiation of angiosperms [71,72,73,74,75,76,77,78]. The Cretaceous is widely recognized as a classic greenhouse period in Earth’s history. Driven by a significant elevation in atmospheric CO_2_ concentrations [79], a global warming trend persisted until approximately 70 Ma. Consequently, we hypothesize that Mantidae originated during the Late Cretaceous, with their diversification process significantly driven by global warming. Ma et al. also suggest that diversity of angiosperms and gymnosperms, as well as atmospheric CO2 concentration, may play critical roles to heterogeneous diversification patterns of predatory insects [80]. This evolutionary pattern has been documented across a diverse range of taxa, including Ephemeroptera, Araneae, Scutigeromorpha, and Caudata [81,82,83,84,85]. Simultaneously, the flourishing of terrestrial vegetation during the Early Jurassic significantly enhanced atmospheric oxygen levels [86]. As broad-spectrum predators, Mantidae possess a diet that encompassed nearly all active small insects of that era. Both fossil and molecular evidence indicate that the diversification bursts of core prey groups coincided with the Cretaceous period, such as Diptera, Orthoptera, and Lepidoptera [87]. Consequently, the predator–prey co-evolution likely played a pivotal role in the radiation and evolutionary development of Mantidae. Furthermore, the synergistic co-evolution among angiosperms, pollinators, and phytophagous insects likely expanded the spectrum of available trophic resources for Mantidae [88]. Notably, the Cretaceous–Paleogene (K-Pg) mass extinction event appears to have had a relatively limited impact on the Mantidae. This resilience can be primarily attributed to their small body sizes, exceptional starvation tolerance, and highly efficient defense and escape strategies, which likely facilitated their survival through this period of extreme climatic upheaval [89].

To ensure transparency, the limitations associated with molecular clock estimations and fossil calibration choices warrant explicit consideration. Mantodea possesses a notoriously sparse and fragmentary fossil record, particularly regarding ancient definitive nodes [90,91,92,93,94]. It can introduce uncertainties and potential node-age biases if priors are not strictly constrained. Specifically, the utilization of *Protohierodula crabbi* [49] as a calibration for Mantidae constraint introduces notable challenges [95]. Because it is an extinct taxon relying on limited morphological synapomorphies preserved in compression fossils—often lacking critical soft-tissue or genital characters—its precise phylogenetic placement remains prone to potential misplacement that can skew divergence time estimates. Consequently, while our study incorporated robust fossil calibrations to anchor key cladogenetic events, the scarcity of well-preserved, stratigraphically constrained fossils highlights the ongoing difficulty of bridging morphological paleontology with molecular phylogenetics. Therefore, divergence time estimates—especially for deeper or rapidly radiating clades—should be interpreted with caution, emphasizing that future discoveries of more complete fossil specimens will be essential to validate these calibration priors and refine the evolutionary timeline of Mantidae.

### 4.4. Historical and Geographical Analysis

By integrating biogeographic distributions with phylogenetic data, we observed that species within the same clades of Mantidae tend to occupy identical or adjacent geographic regions. This congruence suggests that traditional taxonomic and phylogenetic frameworks based solely on morphology are limited by widespread convergent evolution. Consequently, phylogenetic analyses that incorporate both molecular data and geo-ecological distributions offer a more robust and reliable evolutionary reconstruction, and it is consistent with the conclusions of Svenson and Whiting [8].

From the Early Cretaceous to the Late Paleogene, the physical barriers between the southern continental plates were not fully established. Although plates such as Africa, South America, and Australia were trending toward isolation, geological evidence suggests the persistence of long-term and intermittent terrestrial connections among them. The intricacies of these tectonic movements remain immense and poorly understood in many instances. Nonetheless, it is evident that the diversification within Mantidae has been profoundly shaped by these geological events. Although oceanic dispersal is widely recognized as a pivotal factor driving the diversification of numerous insect groups, our biogeographic reconstruction indicates that it played a relatively limited role in the early divergence of the major lineages within Mantidae. Most mantises have flight capabilities sufficient for traversing narrow water barriers, but such dispersal is typically restricted to local geographic scales. Furthermore, mantis oothecae may adhere to driftwood or other flotsam, potentially facilitating transoceanic differentiation through a rafting dispersal model. Although certain mantis possess the potential for flight, females across numerous lineages exhibit pronounced wing reduction and have lost the capacity for sustained long-distance flight [96]. Furthermore, constrained by a significant reduction in wing-beat frequency due to the fusion of basal wing joints, the flight efficiency of most mantis remains low. This dual morphological and physiological constraint strongly supports the view that flight has played only a limited role in the long-distance dispersal of Mantodea on a macroevolutionary scale.

Our ancestral area reconstruction suggests a 51.74% probability that the Mantidae (153 node) originated in the Australasia realms and Neotropical realms at 80.48 Ma, with secondary ancestral probabilities identified in Australasia realms and SE Asia (26.21%). This distribution pattern, centered on the Late Cretaceous, aligns closely with the palaeogeographical configuration of the fragmented Gondwanan landmasses [97]. During the Campanian, Australasia is a region that provided a stable, temperate, and vegetated landscape conducive to early mantid diversification [98,99].

Biogeographic analysis indicates that the Hierodulinae diverged at 64.33 Ma (150 node). Archimantini (126 node) maintained its ancestral distribution in the Australasian realm, while the tribe Hierodulini (149 node) initiated a northward expansion toward Southeast Asia. The stasis exhibited by Archimantini can be attributed to the climatic and structural stability of the Australasian plate during this period. At 64.33 Ma, Australia was characterized by extensive, warm-temperate rainforests dominated by Podocarpaceae and Araucariaceae, which provided a continuous and reliable ecological framework for mantodean persistence [100,101]. Unlike the more volatile tectonic regimes developing along the plate boundaries, the Australasian interior offered a ‘climatic buffer’ under the global greenhouse conditions of the early Paleogene, effectively insulating the Archimantini within a stable refuge [101,102]. Conversely, the northward expansion of Hierodulini was driven by the synergistic tectonic forces of the northward-drifting Australian Plate and the transformative tectonic reconfiguration of the Indian Ocean. As the Australian Plate migrated northward, the collision zone between the Indian, Australian, and Eurasian plates created a transient, archipelagic landscape in the northeastern Indian Ocean—a “stepping-stone” corridor composed of volcanic arcs and microcontinental terranes [97,103].

The emergence of the Stagmomantinae, Mellierinae and Vatinae lineages (118 node) in the Neotropical realm at approximately 61.88 Ma. This divergence time is particularly significant, because it was earlier than the time of the full tectonic separation of South America from the Antarctic–Australasian complex, which occurred progressively during the early Paleogene [104]. We propose that the presence of these lineages in the Neotropics is primarily the result of vicariance coupled with plate drift. As the South American plate migrated westward, ancestral populations were physically separated from their counterparts in the Australasian realm, allowing for independent diversification in the Neotropical ecological environment [105]. This mechanism avoids the necessity of a long-distance marine crossing, because these lineages were essentially taken by the drifting plate, and the process is frequently observed in other Gondwanan insect taxa [106]. Although geographic isolation can explain the initial population differentiation, the trans-Antarctic dispersal model remains an important supplementary research framework [8]. Therefore, it cannot be ruled out that these lineages dispersed from the Australian region to the Neotropical region by a trans-Antarctic route at 61.88 Ma. During the Late Cretaceous, the continuous temperate forests that connected Australia, Antarctica, and South America constituted a key trans-Antarctic migration corridor. Paleoclimatic reconstruction studies based on fossil plants and animals indicate that before these landmasses finally completely separated, this corridor provided conditions for periodic gene flow or range expansion among organisms [107,108].

At the 82 node (71.94 Ma), the common ancestor of Choeradodinae and Orthoderinae occupied a broad range spanning the Australasian and Neotropical realms. Their subsequent paths diverged sharply: Choeradodinae adopted a ‘dispersal-centric’ strategy, exploiting high physiological plasticity to undergo expansive radiation into Southeast Asia and the Neotropics—a trajectory parallel to the global dispersal success of the Vatinae and the opportunistic radiation of Stagmomantinae into the Americas [8,109]. However, the hypothesis of dispersal via the Bering Strait route cannot be ruled out. Because this time coincides with the climax of the Late Cretaceous–Paleocene Boreotropical Flora (BTF) [110]. During this thermal maximum, continuous belts of tropical and subtropical forests at high northern latitudes—connected by the North Atlantic Land Bridge (NALB) and Beringia—facilitated intercontinental biotic exchanges of thermophilic forest-dwelling insects between the Old and New World, such as *Titanomyrma* [111] and Rhinocerotids [112,113].

The emergence of Tenoderinae (101 node) at 58.11 Ma, with a 68.70% probability of origin in the Afrotropical realm and Australasia realms, indicates a rapid establishment in the aftermath of the K-Pg extinction. This initial survival phase laid the foundation for the divergence of the tribe Paramantini (92 node) at 52.03 Ma. While the subtribe Paramantina remained entrenched in the Afrotropical realm, the divergence of the Subtribe Tarachomantina (91 node), which successfully colonized Madagascar, represents a classic instance of sweepstakes dispersal; given the isolation imposed by the Mozambique Channel in the Paleocene, this colonization likely involved rare, long-distance overwater events, which is a mechanism fundamental to the accumulation of island biodiversity [114]. We argue that Tenoderinae benefited from the post-extinction ecological vacuum created by the biotic turnover during the K-Pg transition, which allowed for the rapid colonization of vacant predatory niches as angiosperm-dominated ecosystems expanded [115]. This stability within the Afrotropical cradle allowed for the accumulation of genetic and morphological diversity before the onset of more expansive dispersal pulses in the Paleocene. In this context, we hypothesize that Greater India functioned as a “dynamic ark” for these lineages during the Deccan trap eruptions (66–65 Ma) [115]. While the Deccan volcanism imposed catastrophic environmental stress, the heterogeneous topography of the Indian plate likely created micro-refugia during periods of eruptive quiescence, allowing resilient populations to persist and subsequently benefit from the post-eruptive ecological recovery of angiosperm-dominated ecosystems [116,117]. The radiation of the tribe Tenoderini (100 node) at 44.31 Ma marks a critical shift toward expansive global colonization, inextricably linked to the paleogeographic trajectory of the Indian plate. As India drifted northward and began its collision with the Eurasian margin, it functioned as a vital biogeographic bridge and “evolutionary filter,” facilitating the movement of lineages that had survived the Deccan volcanic pulse toward the Asian continent [97]. This transition allowed the subtribe Tenoderina to penetrate the Palearctic and Southeast Asia, a process highly correlated with the Eocene Climatic Optimum (ECO) [118,119]. Our ancestral area reconstruction indicates that ancestral lineages established within the Palearctic realm. At around 3.52 Ma during the Pliocene, we suggest that the lineages (node 93) expanded across the Beringian northern route into the Nearctic realm. This intercontinental expansion via the high-latitude Beringian pathway aligns with periods of lowered sea levels and regional warming events during the Pliocene, which periodically reconnected northeastern Asia and northwestern North America [119,120].

The ancestor of Mantinae (node 89) retained a 75.93% probability of remaining in the Australasia realms. During the Paleogene, Mantinae started to expand in the Oriental and Palearctic realms. During the early Cenozoic, global warming peaks facilitated the formation of temperate-to-subtropical forest corridors [118]. Mantinae lineages effectively exploited these transient ecological bridges to migrate from their core southern areas into the Eurasian landmass, a process synchronized with major tectonic reorganizations [103].

## 5. Conclusions

This study explores critical phylogenetic uncertainties within Mantidae, confirming the monophyly of Archimantini. Our mitogenomic analyses identify novel gene rearrangements consistent with the TDRL model, providing robust synapomorphies for specific lineages. Divergence dating places the origin of Mantidae in the Late Cretaceous (87.83 Ma), with major subfamily radiation occurring in the Paleocene, coinciding with angiosperm diversification and post-K-Pg recovery. Biogeographic reconstructions support an Australasia realms and Neotropical realms origin, followed by global dispersal via trans-Antarctic land bridges and the “Indian Ark” mechanism. Collectively, these findings offer novel insights into the mechanisms driving the global radiation and diversification of Mantidae, highlighting the profound impact of historical tectonics on their evolutionary trajectory.

## Figures and Tables

**Table 1:** Fossil species used for dating in this study, along with parameters for MCMCTree and references.

Used Fossil Number	Fossils	Bound for MCMCTree	Calibration	Reference
F1	*Baissatermes lapideus*	B(0.943, 4.12)	Cryptocercus + Isoptera	[45]
F2	*Anisotermes xiai*	B(1.37, 4.12)	Mastotermitidae + other Isoptera	[47]
F3	*Arverineura insignis*	L(0.605, 0.1, 0.3)	Chaeteessidae	[50]
F4	*Pseudomantoida extendidera*	L(0.56, 0.1, 0.3)	Mantoididae	[48]
F5	*Protohierodula crabbi*	L(0.34, 0.1, 0.3)	Mantidae	[49]

## Data Availability

Supporting data for this study are available from the National Center for Biotechnology Information (https://www.ncbi.nlm.nih.gov) (accessed on 15 November 2025). The GenBank numbers are PX711349-PX711364.

## Supplementary Materials

The following supporting information can be downloaded at: https://www.mdpi.com/article/10.3390/insects17080830/s1, Figure S1: Circular maps of the 16 mitogenomes; Figure S2: The secondary structure of *tRNA*s in *Pseudomantis albofimbriata* mitogenomes.; Table S1: Species used to construct the phylogenetic relationships along with their sources.; Table S2: Species used to construct the phylogenetic relationships along with GenBank accession numbers.; Table S3: Gene extraction information of each sequence.; Table S4: Ancestral range estimation for dated phylogeny of Mantidae (A: Palearctic, B: Nearctic, C: Neotropical, D: Afrotropical, E: PIP, F: Australasia, G: SE Asia, H: Madagascar).; Table S5: Scores for BioGeoBEARS models.; Table S6: Primers used to verify gene rearrangement.
