# Supplementary material for: A Mitogenome-Based Phylogenetic Framework for Mantidae (Mantodea: Mantoidea): Systematic Implications and Spatio-Temporal Dynamics
Source: Insects. 2026 Aug 10;17(8):830. doi: 10.3390/insects17080830 (PMC13513240; doi:10.3390/insects17080830)
Supplement: Supplementary file 1 [file insects-17-00830-s001.zip › Table S1.pdf]

Table S1 Species used to construct the phylogenetic relationships along with their Sources.

Family	Sucfamily	Tribe	Subtribe	Species	Source
Blattellidae	Blattellinae	Blattellini		*Blattella germanica*	NCBI
Blattidae	Blattinae			*Periplaneta americana*	NCBI
Cryptocercidae				*Cryptocercus relictus*	NCBI
Termitidae	Macrotermitinae			*Macrotermes barneyi*	NCBI
Termitidae	Macrotermitinae			*Mastotermes darwiniensis*	NCBI
Chaeteessidae				*Chaeteessa valida*	Svenson & Whiting(2009)
Deroplatyidae	Deroplatyinae	Deroplatyini	Deroplatyina	*Deroplatys desiccata*	NCBI
Deroplatyidae	Deroplatyinae	Deroplatyini	Deroplatyina	*Deroplatys lobata*	NCBI
Deroplatyidae	Deroplatyinae	Deroplatyini	Deroplatyina	*Deroplatys truncata*	NCBI
Deroplatyidae	Deroplatyinae	Deroplatyini	Pseudempusina	*Pseudempusa pinnapavonis*	NCBI
Deroplatyidae	Deroplatyinae	Euchomenellini		*Euchomenella* sp.	This study
Deroplatyidae	Deroplatyinae	Euchomenellini		*Phasmomantella* sp.	This study
Deroplatyidae	Popinae	Popini		*Neodanuria bolauana*	NCBI
Deroplatyidae	Popinae	Popini		*Popa spurca*	Svenson & Whiting(2009)
Mantidae	Choeradodinae			*Asiadodis yunnanensis*	NCBI
Mantidae	Choeradodinae			*Choeradodis rhombicollis*	Svenson & Whiting(2009)
Mantidae	Choeradodinae			*Choeradodis stalii*	Svenson & Whiting(2009)
Mantidae	Hierodulinae	Archimantini	Archimantina	*Archimantis latistyla*	This study
Mantidae	Hierodulinae	Archimantini	Archimantina	*Archimantis quinquelobata*	This study
Mantidae	Hierodulinae	Archimantini	Archimantina	*Archimantis sobrina*	This study
Mantidae	Hierodulinae	Archimantini	Archimantina	*Austromantis albomarginata*	This study
Mantidae	Hierodulinae	Archimantini	Archimantina	*Austrovates variegata*	This study
Mantidae	Hierodulinae	Archimantini	Pseudomantina	*Pseudomantis albofimbriata*	This study
Mantidae	Hierodulinae	Archimantini	Trachymantina	*Sphodropoda* sp.	Svenson & Whiting(2009)
Mantidae	Hierodulinae	Archimantini	Trachymantina	*Sphodropoda* sp.	This study
Mantidae	Hierodulinae	Hierodulini		*Camelomantis moultoni*	NCBI
Mantidae	Hierodulinae	Hierodulini		*Hierodula chinensis*	NCBI
Mantidae	Hierodulinae	Hierodulini		*Hierodula latipennis*	NCBI
Mantidae	Hierodulinae	Hierodulini		*Hierodula longa*	NCBI
Mantidae	Hierodulinae	Hierodulini		*Hierodula maculata*	NCBI
Mantidae	Hierodulinae	Hierodulini		*Hierodula majuscula*	NCBI
Mantidae	Hierodulinae	Hierodulini		*Hierodula membranacea*	NCBI
Mantidae	Hierodulinae	Hierodulini		*Hierodula multispina*	NCBI
Mantidae	Hierodulinae	Hierodulini		*Hierodula patellifera*	NCBI
Mantidae	Hierodulinae	Hierodulini		*Hierodula pulchripes*	This study
Mantidae	Hierodulinae	Hierodulini		*Hierodula schultzei*	Svenson & Whiting(2009)
Mantidae	Hierodulinae	Hierodulini		*Hierodula* sp.	This study
Mantidae	Hierodulinae	Hierodulini		*Hierodula timorensis*	This study
Mantidae	Hierodulinae	Hierodulini		*Hierodula zhangi*	NCBI
Mantidae	Hierodulinae	Hierodulini		*Ephierodula celebensis*	Svenson & Whiting(2009)
Mantidae	Hierodulinae	Hierodulini		*Ephierodula reticulata*	Svenson & Whiting(2009)

Family	Sucfamily	Tribe	Subtribe	Species	Source
Mantidae	Hierodulinae	Hierodulini		*Pnigomantis medioconstricta*	NCBI
Mantidae	Hierodulinae	Hierodulini		*Rhombodera basalis*	This study
Mantidae	Hierodulinae	Hierodulini		*Rhombodera brachynota*	NCBI
Mantidae	Hierodulinae	Hierodulini		*Rhombodera stalii*	Svenson & Whiting(2009)
Mantidae	Hierodulinae	Hierodulini		*Rhombodera valida*	NCBI
Mantidae	Hierodulinae	Hierodulini		*Tamolanica tamolana*	NCBI
Mantidae	Hierodulinae	Hierodulini		*Tarachomantis alaotrana*	NCBI
Mantidae	Hierodulinae	Hierodulini		*Titanodula formosana*	NCBI
Mantidae	Omomantinae			*Omomantis* sp.	Svenson & Whiting(2009)
Mantidae	Orthoderinae			*Orthodera ministralis*	NCBI
Mantidae	Orthoderinae			*Orthodera novaezealandiae*	This study
Mantidae	Orthoderinae			*Orthodera* sp.	Svenson & Whiting(2009)
Mantidae	Vatinae	Oxyopsidini		*Oxyopsis peruviana*	This study
Mantidae	Vatinae	Oxyopsidini		*Oxyopsis* sp.	Svenson & Whiting(2009)
Mantidae	Vatinae	Oxyopsidini		*Parastagmatoptera* sp.	Svenson & Whiting(2009)
Mantidae	Vatinae	Vatini	Vatina	*Pseudovates chlorophaea*	NCBI
Mantidae	Vatinae	Vatini	Vatina	*Pseudovates cingulata*	Svenson & Whiting(2009)
Mantidae	Vatinae	Vatini	Vatina	*Pseudovates denticulata*	Svenson & Whiting(2009)
Mantidae	Vatinae	Vatini	Vatina	*Pseudovates peruviana*	NCBI
Mantidae	Vatinae	Vatini	Vatina	*Vates pectinacornis*	Svenson & Whiting(2009)
Mantidae	Vatinae	Vatini	Vatina	*Vates sp.*	Svenson & Whiting(2009)
Mantidae	Vatinae	Stagmatopterini		*Stagmatoptera biocellata*	NCBI
Mantidae	Vatinae	Stagmatopterini		*Stagmatoptera* sp.	Svenson & Whiting(2009)
Mantidae	Tenoderinae	Tenoderini	Tenoderina	*Mesopteryx alata*	NCBI
Mantidae	Tenoderinae	Tenoderini	Polyspilotina	*Cataspilota guineensis*	Svenson & Whiting(2009)
Mantidae	Tenoderinae	Tenoderini	Polyspilotina	*Plistospilota* sp.	NCBI
Mantidae	Tenoderinae	Tenoderini	Polyspilotina	*Polyspilota aeruginosa*	Svenson & Whiting(2009)
Mantidae	Tenoderinae	Tenoderini	Polyspilotina	*Prohierodula ornatipennis*	Svenson & Whiting(2009)
Mantidae	Tenoderinae	Tenoderini	Polyspilotina	*Prohierodula picta*	NCBI
Mantidae	Tenoderinae	Tenoderini	Tenoderina	*Tenodera angustipennis*	NCBI
Mantidae	Tenoderinae	Tenoderini	Tenoderina	*Tenodera aridifolia brevicollis*	NCBI
Mantidae	Tenoderinae	Tenoderini	Tenoderina	*Tenodera costalis*	Svenson & Whiting(2009)
Mantidae	Tenoderinae	Tenoderini	Tenoderina	*Tenodera sinensis*	NCBI
Mantidae	Tenoderinae	Paramantini	Paramantina	*Sphodromantis lineola*	NCBI
Mantidae	Tenoderinae	Paramantini	Paramantina	*Sphodromantis viridis*	NCBI
Mantidae	Tenoderinae	Paramantini	Paramantina	*Tarachomantis caldwellii*	Svenson & Whiting(2009)
Mantidae	Stagmomantinae	Antemniini		*Antemna rapax*	Svenson & Whiting(2009)
Mantidae	Stagmomantinae	Stagmomantini		*Phasmomantis* sp.	This study
Mantidae	Stagmomantinae	Stagmomantini		*Stagmomantis carolina*	Svenson & Whiting(2009)
Mantidae	Stagmomantinae	Stagmomantini		*Stagmomantis limbata*	Svenson & Whiting(2009)
Mantidae	Stagmomantinae	Stagmomantini		*Stagmomantis* sp.	Svenson & Whiting(2009)
Mantidae	Stagmomantinae	Stagmomantini		*Stagmomantis vicina*	Svenson & Whiting(2009)
Mantidae	Mantinae			*Mantis religiosa*	NCBI

Family	Sucfamily	Tribe	Subtribe	Species	Source
Mantidae	Mantinae			*Statilia agresta*	NCBI
Mantidae	Mantinae			*Statilia apicalis*	Svenson & Whiting(2009)
Mantidae	Mantinae			*Statilia flavobrunnea*	NCBI
Mantidae	Mantinae			*Statilia maculata*	NCBI
Mantidae	Mantinae			*Statilia nemoralis*	Svenson & Whiting(2009)
Mantidae	Mantinae			*Statilia* sp.	Svenson & Whiting(2009)
Mantidae	Mellierinae	Mellierini		*Xystropeltis* sp.	Svenson & Whiting(2009)
Mantoididae	Mantoididae			*Mantoida schraderi*	Svenson & Whiting(2009)
Mantoididae	Mantoididae			*Mantoida* sp.	Svenson & Whiting(2009)
Mantoididae	Mantoididae			*Mantoida* sp.	Svenson & Whiting(2009)
Mantoididae	Mantoididae			*Mantoida* sp.	Svenson & Whiting(2009)
Mantoididae	Mantoididae			*Mantoida* sp.	Svenson & Whiting(2009)
