# Supplementary material for: A Mitogenome-Based Phylogenetic Framework for Mantidae (Mantodea: Mantoidea): Systematic Implications and Spatio-Temporal Dynamics
Source: Insects. 2026 Aug 10;17(8):830. doi: 10.3390/insects17080830 (PMC13513240; doi:10.3390/insects17080830)
Supplement: Supplementary file 1 [file insects-17-00830-s001.zip › Table S2.pdf]

Table S2 Species used to construct the phylogenetic relationships along with GenBank accession numbers.

Species	No. Accession	12SNo. Accession	16SNo. Accession	COINo. Accession	COIINo. Accession	ND4No. Accession
Antemna rapax	NA	EF383233	EF383393	EF383875	EF384003	FJ802557
Archimantis latistyla	PX711360	NA	NA	NA	NA	NA
Archimantis quinquelobata	PX711354	NA	NA	NA	NA	NA
Archimantis sobrina	PX711350	NA	NA	NA	NA	NA
Asiadodis yunnanensis	MW357297	NA	NA	NA	NA	NA
Austromantis albomarginata	PX711349	NA	NA	NA	NA	NA
Austrovates variegata	PX711357	NA	NA	NA	NA	NA
Blattella germanica	NC_012901	NA	NA	NA	NA	NA
Camelomantis moultoni	KX434812	NA	NA	NA	NA	NA
Cataspilota guineensis	NA	EF383283	EF383441	EF383922	EF384052	FJ802635
Chaeteessa valida	NA	FJ806061	FJ806237	FJ802840	NA	FJ802622
Choeradodis rhombicollis	NA	EF383161	EF383321	EF383805	AY491282	FJ802431
Choeradodis stalii	NA	EF383225	EF383385	EF383868	EF383995	FJ802540
Cryptocercus relictus	JX144941	NA	NA	NA	NA	NA
Deroplatys desiccata	KX434834	NA	NA	NA	NA	NA
Deroplatys lobata	KX434850	NA	NA	NA	NA	NA
Deroplatys truncata	MK400675	NA	NA	NA	NA	NA
Euchomenella sp.	PX711361	NA	NA	NA	NA	NA
Hierodula chinensis	OP108446	NA	NA	NA	NA	NA
Hierodula latipennis	OM202553	NA	NA	NA	NA	NA
Hierodula longa	OK542392	NA	NA	NA	NA	NA
Hierodula maculata	OL414958	NA	NA	NA	NA	NA
Hierodula majuscula	KX434819	NA	NA	NA	NA	NA
Hierodula membranacea	KR703239	NA	NA	NA	NA	NA
Hierodula multispina	KX434830	NA	NA	NA	NA	NA
Hierodula patellifera	KX434853	NA	NA	NA	NA	NA
Hierodula pulchripes	PX711362	NA	NA	NA	NA	NA
Hierodula schultzei	NA	EF383173	EF383333	EF383817	AY491310	FJ802459
Hierodula sp.	PX711356	NA	NA	NA	NA	NA
Hierodula timorensis	PX711359	NA	NA	NA	NA	NA
Hierodula zhangi	MZ673800	NA	NA	NA	NA	NA
Ephierodula celebensis	NA	FJ806133	FJ806316	FJ802914	NA	FJ802739
Ephierodula reticulata	NA	FJ806130	FJ806313	FJ802911	FJ807029	FJ802736
Macrotermes barneyi	JX050221	NA	NA	NA	NA	NA
Mantis religiosa	KU201317	NA	NA	NA	NA	NA
Mantoida schraderi	NA	EF383155	EF383315	EF383799	AY491275	FJ802424

Species	No. Accession	12SNo. Accession	16SNo. Accession	COINo. Accession	COIINo. Accession	ND4No. Accession
Mantoida sp.	NA	FJ806014	FJ806189	FJ802793	FJ806903	FJ802523
Mantoida sp.	NA	FJ806015	FJ806190	FJ802794	FJ806904	FJ802524
Mantoida sp.	NA	EF383249	EF383408	EF383890	EF384019	FJ802585
Mantoida sp.	NA	FJ806044	FJ806220	FJ802822	FJ806935	FJ802586
Mastotermes darwiniensis	OM646554	NA	NA	NA	NA	NA
Mesopteryx alata	KX434813	NA	NA	NA	NA	NA
Neodanuria bolauana	KX434858	NA	NA	NA	NA	NA
Omomantis sp.	NA	EF383304	EF383463	EF383944	EF384075	FJ802671
Orthodera ministralis	KX434842	NA	NA	NA	NA	NA
Orthodera novaezealandiae	NA	NA	NA	NA	NA	NA
Orthodera sp.	NA	FJ805987	FJ806163	FJ802766	AY491299	FJ802448
Oxyopsis peruviana	PX711353	NA	NA	NA	NA	NA
Oxyopsis sp.	NA	FJ806094	FJ806267	FJ802868	FJ806983	FJ802689
Parastagmatoptera sp.	NA	FJ805983	FJ806159	FJ802762	AY491294	FJ802443
Periplaneta americana	NC016956	NA	NA	NA	NA	NA
Phasmomantella sp.	PX711352	NA	NA	NA	NA	NA
Phasmomantis sp.	PX711364	NA	NA	NA	NA	NA
Plistospilota sp.	KX434821	NA	NA	NA	NA	NA
Pnigomantis medioconstricta	KX434851	NA	NA	NA	NA	NA
Polyspilota aeruginosa	NA	FJ806068	FJ806244	FJ802847	FJ806958	FJ802647
Popa spurca	NA	EF383227	EF383387	EF383870	EF383997	FJ802546
Prohierodula ornatipennis	NA	EF383291	EF383449	EF383930	EF384060	FJ802648
Prohierodula picta	KX434855	NA	NA	NA	NA	NA
Pseudempusa pinnapavonis	OM202557	NA	NA	NA	NA	NA
Pseudomantis albofimbriata	PX711358	NA	NA	NA	NA	NA
Pseudovates chlorophaea	KX434838	NA	NA	NA	NA	NA
Pseudovates cingulata	NA	FJ806060	FJ806236	FJ802839	FJ806951	FJ802619
Pseudovates denticulata	NA	NA	FJ806283	FJ802882	FJ807000	FJ802706
Pseudovates peruviana	MW357301	NA	NA	NA	NA	NA
Rhombodera basalis	PX711363	NA	NA	NA	NA	NA
Rhombodera brachynota	KX611802	NA	NA	NA	NA	NA
Rhombodera stalii	NA	EF383162	EF383322	EF383806	AY491284	FJ802433
Rhombodera valida	KX611804	NA	NA	NA	NA	NA
Sphodromantis lineola	KY689123	NA	NA	NA	NA	NA
Sphodromantis viridis	KX434840	NA	NA	NA	NA	NA
Sphodropoda sp.	NA	EF383303	EF383462	EF383943	EF384074	FJ802670

Species	No. Accession	12SNo. Accession	16SNo. Accession	COINo. Accession	COIINo. Accession	ND4No. Accession
Sphodropoda sp.	PX711351	NA	NA	NA	NA	NA
Stagmatoptera biocellata	MW357302	NA	NA	NA	NA	NA
Stagmatoptera sp.	NA	EF383220	EF383380	EF383863	EF383990	FJ802531
Stagmomantis carolina	NA	EF383165	EF383325	EF383809	AY491289	FJ802438
Stagmomantis limbata	NA	EF383168	EF383328	EF383812	AY491297	FJ802446
Stagmomantis sp.	NA	FJ805982	FJ806158	FJ802761	AY491293	FJ802442
Stagmomantis vicina	NA	FJ805981	FJ806157	FJ802760	AY491290	FJ802439
Statilia agresta	OM202556	NA	NA	NA	NA	NA
Statilia apicalis	NA	FJ805995	FJ806171	FJ802774	AY491313	FJ802463
Statilia flavobrunnea	OK542391	NA	NA	NA	NA	NA
Statilia maculata	KX434809	NA	NA	NA	NA	NA
Statilia nemoralis	NA	FJ806002	FJ806177	FJ802781	FJ806891	FJ802493
Statilia sp.	NA	FJ806070	FJ806246	FJ802849	FJ806960	FJ802653
Tamolanica tamolana	NC007702	NA	NA	NA	NA	NA
Tarachomantis alaotrana	KX434862	NA	NA	NA	NA	NA
Tarachomantis caldwellii	NA	NA	FJ806281	FJ802880	FJ806998	FJ802704
Tenodera angustipennis	KX434852	NA	NA	NA	NA	NA
Tenodera aridifolia brevicollis	OK542393	NA	NA	NA	NA	NA
Tenodera costalis	NA	EF383166	EF383326	EF383810	AY491291	FJ802440
Tenodera sinensis	KX673199	NA	NA	NA	NA	NA
Titanodula formosana	KR703238	NA	NA	NA	NA	NA
Vates pectinacornis	NA	EF383159	EF383319	EF383803	AY491280	FJ802429
Vates sp.	NA	FJ806019	FJ806194	FJ802798	FJ806908	FJ802532
Xystropeltis sp.	NA	EF383299	EF383459	NA	EF384070	FJ802664
