# Supplementary material for: A Mitogenome-Based Phylogenetic Framework for Mantidae (Mantodea: Mantoidea): Systematic Implications and Spatio-Temporal Dynamics
Source: Insects. 2026 Aug 10;17(8):830. doi: 10.3390/insects17080830 (PMC13513240; doi:10.3390/insects17080830)
Supplement: Supplementary file 1 [file insects-17-00830-s001.zip › Table S3.pdf]

Table S3 Gene extraction information of each sequence

Species	Gene	Status	Accession	Length(bp)
Antemna rapax	12S	Extracted	EF383233	378
Antemna rapax	16S	Extracted	EF383393	565
Antemna rapax	ATP6	Missing	NA	0
Antemna rapax	ATP8	Missing	NA	0
Antemna rapax	COX1	Extracted	EF383875	1399
Antemna rapax	COX2	Extracted	EF384003	636
Antemna rapax	COX3	Missing	NA	0
Antemna rapax	CYTB	Missing	NA	0
Antemna rapax	ND1	Missing	NA	0
Antemna rapax	ND2	Missing	NA	0
Antemna rapax	ND3	Missing	NA	0
Antemna rapax	ND4	Extracted	FJ802557	1256
Antemna rapax	ND4L	Missing	NA	0
Antemna rapax	ND5	Missing	NA	0
Antemna rapax	ND6	Missing	NA	0
Archimantis latistyla	12S	Extracted	BXOZ	794
Archimantis latistyla	16S	Extracted	BXOZ	1314
Archimantis latistyla	ATP6	Extracted	BXOZ	681
Archimantis latistyla	ATP8	Extracted	BXOZ	159
Archimantis latistyla	COX1	Extracted	BXOZ	1538
Archimantis latistyla	COX2	Extracted	BXOZ	685
Archimantis latistyla	COX3	Extracted	BXOZ	789
Archimantis latistyla	CYTB	Extracted	BXOZ	1134
Archimantis latistyla	ND1	Extracted	BXOZ	933
Archimantis latistyla	ND2	Extracted	BXOZ	1029
Archimantis latistyla	ND3	Extracted	BXOZ	354
Archimantis latistyla	ND4	Extracted	BXOZ	1335
Archimantis latistyla	ND4L	Extracted	BXOZ	282
Archimantis latistyla	ND5	Extracted	BXOZ	1724
Archimantis latistyla	ND6	Extracted	BXOZ	504
Archimantis quinquelobata	12S	Extracted	WYST	784
Archimantis quinquelobata	16S	Extracted	WYST	1309
Archimantis quinquelobata	ATP6	Extracted	WYST	681
Archimantis quinquelobata	ATP8	Extracted	WYST	159
Archimantis quinquelobata	COX1	Extracted	WYST	1538
Archimantis quinquelobata	COX2	Extracted	WYST	685
Archimantis quinquelobata	COX3	Extracted	WYST	789
Archimantis quinquelobata	CYTB	Extracted	WYST	1140
Archimantis quinquelobata	ND1	Extracted	WYST	939
Archimantis quinquelobata	ND2	Extracted	WYST	1029
Archimantis quinquelobata	ND3	Extracted	WYST	354

Species	Gene	Status	Accession	Length(bp)
Ar*chimantis quinquelobata*	ND4	Extracted	WYST	1338
Ar*chimantis quinquelobata*	ND4L	Extracted	WYST	282
Ar*chimantis quinquelobata*	ND5	Extracted	WYST	1724
Ar*chimantis quinquelobata*	ND6	Extracted	WYST	504
Ar*chimantis sobrina*	12S	Extracted	WZST	794
Ar*chimantis sobrina*	16S	Extracted	WZST	1314
Ar*chimantis sobrina*	ATP6	Extracted	WZST	681
Ar*chimantis sobrina*	ATP8	Extracted	WZST	159
Ar*chimantis sobrina*	COX1	Extracted	WZST	1538
Ar*chimantis sobrina*	COX2	Extracted	WZST	685
Ar*chimantis sobrina*	COX3	Extracted	WZST	789
Ar*chimantis sobrina*	CYTB	Extracted	WZST	1134
Ar*chimantis sobrina*	ND1	Extracted	WZST	933
Ar*chimantis sobrina*	ND2	Extracted	WZST	1008
Ar*chimantis sobrina*	ND3	Extracted	WZST	354
Ar*chimantis sobrina*	ND4	Extracted	WZST	1335
Ar*chimantis sobrina*	ND4L	Extracted	WZST	282
Ar*chimantis sobrina*	ND5	Extracted	WZST	1724
Ar*chimantis sobrina*	ND6	Extracted	WZST	504
As*iadodis yunnanensis*	12S	Extracted	MW357297	790
As*iadodis yunnanensis*	16S	Extracted	MW357297	1318
As*iadodis yunnanensis*	ATP6	Extracted	MW357297	678
As*iadodis yunnanensis*	ATP8	Extracted	MW357297	159
As*iadodis yunnanensis*	COX1	Extracted	MW357297	1536
As*iadodis yunnanensis*	COX2	Extracted	MW357297	685
As*iadodis yunnanensis*	COX3	Extracted	MW357297	789
As*iadodis yunnanensis*	CYTB	Extracted	MW357297	1137
As*iadodis yunnanensis*	ND1	Extracted	MW357297	936
As*iadodis yunnanensis*	ND2	Extracted	MW357297	1029
As*iadodis yunnanensis*	ND3	Extracted	MW357297	354
As*iadodis yunnanensis*	ND4	Extracted	MW357297	1338
As*iadodis yunnanensis*	ND4L	Extracted	MW357297	282
As*iadodis yunnanensis*	ND5	Extracted	MW357297	1723
As*iadodis yunnanensis*	ND6	Extracted	MW357297	504
Au*stromantis albomarginata*	12S	Extracted	OZBY	797
Au*stromantis albomarginata*	16S	Extracted	OZBY	1325
Au*stromantis albomarginata*	ATP6	Extracted	OZBY	681
Au*stromantis albomarginata*	ATP8	Extracted	OZBY	159
Au*stromantis albomarginata*	COX1	Extracted	OZBY	1536
Au*stromantis albomarginata*	COX2	Extracted	OZBY	685
Au*stromantis albomarginata*	COX3	Extracted	OZBY	789
Au*stromantis albomarginata*	CYTB	Extracted	OZBY	1134
Au*stromantis albomarginata*	ND1	Extracted	OZBY	936

Species	Gene	Status	Accession	Length(bp)
Austromantis albomarginata	ND2	Extracted	OZBY	1026
Austromantis albomarginata	ND3	Extracted	OZBY	354
Austromantis albomarginata	ND4	Extracted	OZBY	1338
Austromantis albomarginata	ND4L	Extracted	OZBY	282
Austromantis albomarginata	ND5	Extracted	OZBY	1749
Austromantis albomarginata	ND6	Extracted	OZBY	504
Austrovates variegata	12S	Extracted	OZNY	797
Austrovates variegata	16S	Extracted	OZNY	1325
Austrovates variegata	ATP6	Extracted	OZNY	678
Austrovates variegata	ATP8	Extracted	OZNY	159
Austrovates variegata	COX1	Extracted	OZNY	1536
Austrovates variegata	COX2	Extracted	OZNY	685
Austrovates variegata	COX3	Extracted	OZNY	789
Austrovates variegata	CYTB	Extracted	OZNY	1134
Austrovates variegata	ND1	Extracted	OZNY	936
Austrovates variegata	ND2	Extracted	OZNY	1026
Austrovates variegata	ND3	Extracted	OZNY	354
Austrovates variegata	ND4	Extracted	OZNY	1338
Austrovates variegata	ND4L	Extracted	OZNY	282
Austrovates variegata	ND5	Extracted	OZNY	1723
Austrovates variegata	ND6	Extracted	OZNY	504
Blattella germanica	12S	Extracted	NC 012901	794
Blattella germanica	16S	Extracted	NC 012901	1308
Blattella germanica	ATP6	Extracted	NC 012901	681
Blattella germanica	ATP8	Extracted	NC 012901	159
Blattella germanica	COX1	Extracted	NC 012901	1536
Blattella germanica	COX2	Extracted	NC 012901	685
Blattella germanica	COX3	Extracted	NC 012901	789
Blattella germanica	CYTB	Extracted	NC 012901	1134
Blattella germanica	ND1	Extracted	NC 012901	942
Blattella germanica	ND2	Extracted	NC 012901	1026
Blattella germanica	ND3	Extracted	NC 012901	354
Blattella germanica	ND4	Extracted	NC 012901	1340
Blattella germanica	ND4L	Extracted	NC 012901	282
Blattella germanica	ND5	Extracted	NC 012901	1716
Blattella germanica	ND6	Extracted	NC 012901	501
Camelomantis moultoni	12S	Extracted	KX434812	777
Camelomantis moultoni	16S	Extracted	KX434812	1319
Camelomantis moultoni	ATP6	Extracted	KX434812	678
Camelomantis moultoni	ATP8	Extracted	KX434812	159
Camelomantis moultoni	COX1	Extracted	KX434812	1536
Camelomantis moultoni	COX2	Extracted	KX434812	685
Camelomantis moultoni	COX3	Extracted	KX434812	789

Species	Gene	Status	Accession	Length(bp)
Camelomantis moultoni	CYTB	Extracted	KX434812	1133
Camelomantis moultoni	ND1	Extracted	KX434812	936
Camelomantis moultoni	ND2	Extracted	KX434812	1026
Camelomantis moultoni	ND3	Extracted	KX434812	354
Camelomantis moultoni	ND4	Extracted	KX434812	1338
Camelomantis moultoni	ND4L	Extracted	KX434812	282
Camelomantis moultoni	ND5	Extracted	KX434812	1723
Camelomantis moultoni	ND6	Extracted	KX434812	504
Cataspilota guineensis	12S	Extracted	EF383283	376
Cataspilota guineensis	16S	Extracted	EF383441	561
Cataspilota guineensis	ATP6	Missing	NA	0
Cataspilota guineensis	ATP8	Missing	NA	0
Cataspilota guineensis	COX1	Extracted	EF383922	1414
Cataspilota guineensis	COX2	Extracted	EF384052	636
Cataspilota guineensis	COX3	Missing	NA	0
Cataspilota guineensis	CYTB	Missing	NA	0
Cataspilota guineensis	ND1	Missing	NA	0
Cataspilota guineensis	ND2	Missing	NA	0
Cataspilota guineensis	ND3	Missing	NA	0
Cataspilota guineensis	ND4	Extracted	FJ802635	1278
Cataspilota guineensis	ND4L	Missing	NA	0
Cataspilota guineensis	ND5	Missing	NA	0
Cataspilota guineensis	ND6	Missing	NA	0
Chaeteessa valida	12S	Extracted	FJ806061	393
Chaeteessa valida	16S	Extracted	FJ806237	524
Chaeteessa valida	ATP6	Missing	NA	0
Chaeteessa valida	ATP8	Missing	NA	0
Chaeteessa valida	COX1	Extracted	FJ802840	495
Chaeteessa valida	COX2	Missing	NA	0
Chaeteessa valida	COX3	Missing	NA	0
Chaeteessa valida	CYTB	Missing	NA	0
Chaeteessa valida	ND1	Missing	NA	0
Chaeteessa valida	ND2	Missing	NA	0
Chaeteessa valida	ND3	Missing	NA	0
Chaeteessa valida	ND4	Extracted	FJ802622	1261
Chaeteessa valida	ND4L	Missing	NA	0
Chaeteessa valida	ND5	Missing	NA	0
Chaeteessa valida	ND6	Missing	NA	0
Choeradodis rhombicollis	12S	Extracted	EF383161	378
Choeradodis rhombicollis	16S	Extracted	EF383321	550
Choeradodis rhombicollis	ATP6	Missing	NA	0
Choeradodis rhombicollis	ATP8	Missing	NA	0
Choeradodis rhombicollis	COX1	Extracted	EF383805	1414

Species	Gene	Status	Accession	Length(bp)
Choeradodis rhombicollis	COX2	Extracted	AY491282	563
Choeradodis rhombicollis	COX3	Missing	NA	0
Choeradodis rhombicollis	CYTB	Missing	NA	0
Choeradodis rhombicollis	ND1	Missing	NA	0
Choeradodis rhombicollis	ND2	Missing	NA	0
Choeradodis rhombicollis	ND3	Missing	NA	0
Choeradodis rhombicollis	ND4	Extracted	FJ802431	1278
Choeradodis rhombicollis	ND4L	Missing	NA	0
Choeradodis rhombicollis	ND5	Missing	NA	0
Choeradodis rhombicollis	ND6	Missing	NA	0
Choeradodis stalii	12S	Extracted	EF383225	377
Choeradodis stalii	16S	Extracted	EF383385	557
Choeradodis stalii	ATP6	Missing	NA	0
Choeradodis stalii	ATP8	Missing	NA	0
Choeradodis stalii	COX1	Extracted	EF383868	1400
Choeradodis stalii	COX2	Extracted	EF383995	636
Choeradodis stalii	COX3	Missing	NA	0
Choeradodis stalii	CYTB	Missing	NA	0
Choeradodis stalii	ND1	Missing	NA	0
Choeradodis stalii	ND2	Missing	NA	0
Choeradodis stalii	ND3	Missing	NA	0
Choeradodis stalii	ND4	Extracted	FJ802540	1278
Choeradodis stalii	ND4L	Missing	NA	0
Choeradodis stalii	ND5	Missing	NA	0
Choeradodis stalii	ND6	Missing	NA	0
Cryptocercus relictus	12S	Extracted	JX144941	782
Cryptocercus relictus	16S	Extracted	JX144941	1281
Cryptocercus relictus	ATP6	Extracted	JX144941	681
Cryptocercus relictus	ATP8	Extracted	JX144941	159
Cryptocercus relictus	COX1	Extracted	JX144941	1536
Cryptocercus relictus	COX2	Extracted	JX144941	685
Cryptocercus relictus	COX3	Extracted	JX144941	789
Cryptocercus relictus	CYTB	Extracted	JX144941	1132
Cryptocercus relictus	ND1	Extracted	JX144941	964
Cryptocercus relictus	ND2	Extracted	JX144941	1028
Cryptocercus relictus	ND3	Extracted	JX144941	353
Cryptocercus relictus	ND4	Extracted	JX144941	1341
Cryptocercus relictus	ND4L	Extracted	JX144941	288
Cryptocercus relictus	ND5	Extracted	JX144941	1729
Cryptocercus relictus	ND6	Extracted	JX144941	498
Deroplatys desiccata	12S	Extracted	KX434834	781
Deroplatys desiccata	16S	Extracted	KX434834	1330
Deroplatys desiccata	ATP6	Extracted	KX434834	681

Species	Gene	Status	Accession	Length(bp)
Deroplatys desiccata	ATP8	Extracted	KX434834	162
Deroplatys desiccata	COX1	Extracted	KX434834	1557
Deroplatys desiccata	COX2	Extracted	KX434834	684
Deroplatys desiccata	COX3	Extracted	KX434834	789
Deroplatys desiccata	CYTB	Extracted	KX434834	1134
Deroplatys desiccata	ND1	Extracted	KX434834	936
Deroplatys desiccata	ND2	Extracted	KX434834	1032
Deroplatys desiccata	ND3	Extracted	KX434834	354
Deroplatys desiccata	ND4	Extracted	KX434834	1338
Deroplatys desiccata	ND4L	Extracted	KX434834	282
Deroplatys desiccata	ND5	Extracted	KX434834	1720
Deroplatys desiccata	ND6	Extracted	KX434834	516
Deroplatys lobata	12S	Extracted	KX434850	782
Deroplatys lobata	16S	Extracted	KX434850	1306
Deroplatys lobata	ATP6	Extracted	KX434850	678
Deroplatys lobata	ATP8	Extracted	KX434850	159
Deroplatys lobata	COX1	Extracted	KX434850	1542
Deroplatys lobata	COX2	Extracted	KX434850	685
Deroplatys lobata	COX3	Extracted	KX434850	791
Deroplatys lobata	CYTB	Extracted	KX434850	1137
Deroplatys lobata	ND1	Extracted	KX434850	936
Deroplatys lobata	ND2	Extracted	KX434850	1035
Deroplatys lobata	ND3	Extracted	KX434850	354
Deroplatys lobata	ND4	Extracted	KX434850	1338
Deroplatys lobata	ND4L	Extracted	KX434850	285
Deroplatys lobata	ND5	Extracted	KX434850	1723
Deroplatys lobata	ND6	Extracted	KX434850	504
Deroplatys truncata	12S	Extracted	MK400675	782
Deroplatys truncata	16S	Extracted	MK400675	1309
Deroplatys truncata	ATP6	Extracted	MK400675	678
Deroplatys truncata	ATP8	Extracted	MK400675	156
Deroplatys truncata	COX1	Extracted	MK400675	1536
Deroplatys truncata	COX2	Extracted	MK400675	691
Deroplatys truncata	COX3	Extracted	MK400675	792
Deroplatys truncata	CYTB	Extracted	MK400675	1134
Deroplatys truncata	ND1	Extracted	MK400675	936
Deroplatys truncata	ND2	Extracted	MK400675	1026
Deroplatys truncata	ND3	Extracted	MK400675	354
Deroplatys truncata	ND4	Extracted	MK400675	1314
Deroplatys truncata	ND4L	Extracted	MK400675	279
Deroplatys truncata	ND5	Extracted	MK400675	1720
Deroplatys truncata	ND6	Extracted	MK400675	504
Euchomenella sp.	12S	Extracted	MLCJ	808

Species	Gene	Status	Accession	Length(bp)
Eu*chomenella* sp.	16S	Extracted	MLCJ	1333
Eu*chomenella* sp.	ATP6	Extracted	MLCJ	678
Eu*chomenella* sp.	ATP8	Extracted	MLCJ	162
Eu*chomenella* sp.	COX1	Extracted	MLCJ	1536
Eu*chomenella* sp.	COX2	Extracted	MLCJ	688
Eu*chomenella* sp.	COX3	Extracted	MLCJ	789
Eu*chomenella* sp.	CYTB	Extracted	MLCJ	1137
Eu*chomenella* sp.	ND1	Extracted	MLCJ	936
Eu*chomenella* sp.	ND2	Extracted	MLCJ	1026
Eu*chomenella* sp.	ND3	Extracted	MLCJ	354
Eu*chomenella* sp.	ND4	Extracted	MLCJ	1335
Eu*chomenella* sp.	ND4L	Extracted	MLCJ	282
Eu*chomenella* sp.	ND5	Extracted	MLCJ	1724
Eu*chomenella* sp.	ND6	Extracted	MLCJ	510
Hi*erodula chinensis*	12S	Extracted	OP108446	809
Hi*erodula chinensis*	16S	Extracted	OP108446	1315
Hi*erodula chinensis*	ATP6	Extracted	OP108446	678
Hi*erodula chinensis*	ATP8	Extracted	OP108446	159
Hi*erodula chinensis*	COX1	Extracted	OP108446	1536
Hi*erodula chinensis*	COX2	Extracted	OP108446	685
Hi*erodula chinensis*	COX3	Extracted	OP108446	792
Hi*erodula chinensis*	CYTB	Extracted	OP108446	1137
Hi*erodula chinensis*	ND1	Extracted	OP108446	936
Hi*erodula chinensis*	ND2	Extracted	OP108446	1029
Hi*erodula chinensis*	ND3	Extracted	OP108446	354
Hi*erodula chinensis*	ND4	Extracted	OP108446	1335
Hi*erodula chinensis*	ND4L	Extracted	OP108446	282
Hi*erodula chinensis*	ND5	Extracted	OP108446	1740
Hi*erodula chinensis*	ND6	Extracted	OP108446	501
Hi*erodula latipennis*	12S	Extracted	OM202553	800
Hi*erodula latipennis*	16S	Extracted	OM202553	1320
Hi*erodula latipennis*	ATP6	Extracted	OM202553	681
Hi*erodula latipennis*	ATP8	Extracted	OM202553	159
Hi*erodula latipennis*	COX1	Extracted	OM202553	1536
Hi*erodula latipennis*	COX2	Extracted	OM202553	685
Hi*erodula latipennis*	COX3	Extracted	OM202553	789
Hi*erodula latipennis*	CYTB	Extracted	OM202553	1137
Hi*erodula latipennis*	ND1	Extracted	OM202553	936
Hi*erodula latipennis*	ND2	Extracted	OM202553	1029
Hi*erodula latipennis*	ND3	Extracted	OM202553	354
Hi*erodula latipennis*	ND4	Extracted	OM202553	1338
Hi*erodula latipennis*	ND4L	Extracted	OM202553	282
Hi*erodula latipennis*	ND5	Extracted	OM202553	1723

Species	Gene	Status	Accession	Length(bp)
Hierodula latipennis	ND6	Extracted	OM202553	504
Hierodula longa	12S	Extracted	OK542392	790
Hierodula longa	16S	Extracted	OK542392	1313
Hierodula longa	ATP6	Extracted	OK542392	681
Hierodula longa	ATP8	Extracted	OK542392	159
Hierodula longa	COX1	Extracted	OK542392	1536
Hierodula longa	COX2	Extracted	OK542392	685
Hierodula longa	COX3	Extracted	OK542392	789
Hierodula longa	CYTB	Extracted	OK542392	1137
Hierodula longa	ND1	Extracted	OK542392	936
Hierodula longa	ND2	Extracted	OK542392	1029
Hierodula longa	ND3	Extracted	OK542392	354
Hierodula longa	ND4	Extracted	OK542392	1338
Hierodula longa	ND4L	Extracted	OK542392	282
Hierodula longa	ND5	Extracted	OK542392	1723
Hierodula longa	ND6	Extracted	OK542392	504
Hierodula maculata	12S	Extracted	OL414958	798
Hierodula maculata	16S	Extracted	OL414958	1319
Hierodula maculata	ATP6	Extracted	OL414958	681
Hierodula maculata	ATP8	Extracted	OL414958	159
Hierodula maculata	COX1	Extracted	OL414958	1536
Hierodula maculata	COX2	Extracted	OL414958	685
Hierodula maculata	COX3	Extracted	OL414958	789
Hierodula maculata	CYTB	Extracted	OL414958	1137
Hierodula maculata	ND1	Extracted	OL414958	936
Hierodula maculata	ND2	Extracted	OL414958	1029
Hierodula maculata	ND3	Extracted	OL414958	354
Hierodula maculata	ND4	Extracted	OL414958	1338
Hierodula maculata	ND4L	Extracted	OL414958	282
Hierodula maculata	ND5	Extracted	OL414958	1723
Hierodula maculata	ND6	Extracted	OL414958	504
Hierodula majuscula	12S	Extracted	KX434819	780
Hierodula majuscula	16S	Extracted	KX434819	1316
Hierodula majuscula	ATP6	Extracted	KX434819	678
Hierodula majuscula	ATP8	Extracted	KX434819	159
Hierodula majuscula	COX1	Extracted	KX434819	1539
Hierodula majuscula	COX2	Extracted	KX434819	685
Hierodula majuscula	COX3	Extracted	KX434819	795
Hierodula majuscula	CYTB	Extracted	KX434819	1134
Hierodula majuscula	ND1	Extracted	KX434819	936
Hierodula majuscula	ND2	Extracted	KX434819	1029
Hierodula majuscula	ND3	Extracted	KX434819	354
Hierodula majuscula	ND4	Extracted	KX434819	1341

Species	Gene	Status	Accession	Length(bp)
Hierodula majuscula	ND4L	Extracted	KX434819	282
Hierodula majuscula	ND5	Extracted	KX434819	1723
Hierodula majuscula	ND6	Extracted	KX434819	504
Hierodula membranacea	12S	Extracted	KR703239	773
Hierodula membranacea	16S	Extracted	KR703239	1310
Hierodula membranacea	ATP6	Extracted	KR703239	678
Hierodula membranacea	ATP8	Extracted	KR703239	159
Hierodula membranacea	COX1	Extracted	KR703239	1539
Hierodula membranacea	COX2	Extracted	KR703239	685
Hierodula membranacea	COX3	Extracted	KR703239	792
Hierodula membranacea	CYTB	Extracted	KR703239	1134
Hierodula membranacea	ND1	Extracted	KR703239	936
Hierodula membranacea	ND2	Extracted	KR703239	1027
Hierodula membranacea	ND3	Extracted	KR703239	354
Hierodula membranacea	ND4	Extracted	KR703239	1338
Hierodula membranacea	ND4L	Extracted	KR703239	282
Hierodula membranacea	ND5	Extracted	KR703239	1720
Hierodula membranacea	ND6	Extracted	KR703239	504
Hierodula multispina	12S	Extracted	KX434830	777
Hierodula multispina	16S	Extracted	KX434830	1315
Hierodula multispina	ATP6	Extracted	KX434830	681
Hierodula multispina	ATP8	Extracted	KX434830	159
Hierodula multispina	COX1	Extracted	KX434830	1539
Hierodula multispina	COX2	Extracted	KX434830	685
Hierodula multispina	COX3	Extracted	KX434830	792
Hierodula multispina	CYTB	Extracted	KX434830	1137
Hierodula multispina	ND1	Extracted	KX434830	936
Hierodula multispina	ND2	Extracted	KX434830	1029
Hierodula multispina	ND3	Extracted	KX434830	354
Hierodula multispina	ND4	Extracted	KX434830	1338
Hierodula multispina	ND4L	Extracted	KX434830	282
Hierodula multispina	ND5	Extracted	KX434830	1723
Hierodula multispina	ND6	Extracted	KX434830	504
Hierodula patellifera	12S	Extracted	KX434853	777
Hierodula patellifera	16S	Extracted	KX434853	1316
Hierodula patellifera	ATP6	Extracted	KX434853	678
Hierodula patellifera	ATP8	Extracted	KX434853	159
Hierodula patellifera	COX1	Extracted	KX434853	1539
Hierodula patellifera	COX2	Extracted	KX434853	685
Hierodula patellifera	COX3	Extracted	KX434853	792
Hierodula patellifera	CYTB	Extracted	KX434853	1137
Hierodula patellifera	ND1	Extracted	KX434853	936
Hierodula patellifera	ND2	Extracted	KX434853	1029

Species	Gene	Status	Accession	Length(bp)
Hierodula patellifera	ND3	Extracted	KX434853	354
Hierodula patellifera	ND4	Extracted	KX434853	1338
Hierodula patellifera	ND4L	Extracted	KX434853	282
Hierodula patellifera	ND5	Extracted	KX434853	1720
Hierodula patellifera	ND6	Extracted	KX434853	504
Hierodula pulchripes	12S	Extracted	YZFT	793
Hierodula pulchripes	16S	Extracted	YZFT	1318
Hierodula pulchripes	ATP6	Extracted	YZFT	678
Hierodula pulchripes	ATP8	Extracted	YZFT	159
Hierodula pulchripes	COX1	Extracted	YZFT	1536
Hierodula pulchripes	COX2	Extracted	YZFT	685
Hierodula pulchripes	COX3	Extracted	YZFT	792
Hierodula pulchripes	CYTB	Extracted	YZFT	1134
Hierodula pulchripes	ND1	Extracted	YZFT	936
Hierodula pulchripes	ND2	Extracted	YZFT	1029
Hierodula pulchripes	ND3	Extracted	YZFT	354
Hierodula pulchripes	ND4	Extracted	YZFT	1338
Hierodula pulchripes	ND4L	Extracted	YZFT	282
Hierodula pulchripes	ND5	Extracted	YZFT	1720
Hierodula pulchripes	ND6	Extracted	YZFT	504
Hierodula schultzei	12S	Extracted	EF383173	376
Hierodula schultzei	16S	Extracted	EF383333	550
Hierodula schultzei	ATP6	Missing	NA	0
Hierodula schultzei	ATP8	Missing	NA	0
Hierodula schultzei	COX1	Extracted	EF383817	1411
Hierodula schultzei	COX2	Extracted	AY491310	563
Hierodula schultzei	COX3	Missing	NA	0
Hierodula schultzei	CYTB	Missing	NA	0
Hierodula schultzei	ND1	Missing	NA	0
Hierodula schultzei	ND2	Missing	NA	0
Hierodula schultzei	ND3	Missing	NA	0
Hierodula schultzei	ND4	Extracted	FJ802459	1278
Hierodula schultzei	ND4L	Missing	NA	0
Hierodula schultzei	ND5	Missing	NA	0
Hierodula schultzei	ND6	Missing	NA	0
Hierodula sp.	12S	Extracted	YNHJFT01	800
Hierodula sp.	16S	Extracted	YNHJFT01	1311
Hierodula sp.	ATP6	Extracted	YNHJFT01	681
Hierodula sp.	ATP8	Extracted	YNHJFT01	159
Hierodula sp.	COX1	Extracted	YNHJFT01	1536
Hierodula sp.	COX2	Extracted	YNHJFT01	685
Hierodula sp.	COX3	Extracted	YNHJFT01	789
Hierodula sp.	CYTB	Extracted	YNHJFT01	1137

Species	Gene	Status	Accession	Length(bp)
Hierodula sp.	ND1	Extracted	YNHJFT01	936
Hierodula sp.	ND2	Extracted	YNHJFT01	1029
Hierodula sp.	ND3	Extracted	YNHJFT01	354
Hierodula sp.	ND4	Extracted	YNHJFT01	1338
Hierodula sp.	ND4L	Extracted	YNHJFT01	282
Hierodula sp.	ND5	Extracted	YNHJFT01	1723
Hierodula sp.	ND6	Extracted	YNHJFT01	504
Hierodula timorensis	12S	Extracted	DWFT	793
Hi*erodula timorensis*	16S	Extracted	DWFT	1312
Hi*erodula timorensis*	ATP6	Extracted	DWFT	678
Hi*erodula timorensis*	ATP8	Extracted	DWFT	159
Hi*erodula timorensis*	COX1	Extracted	DWFT	1539
Hi*erodula timorensis*	COX2	Extracted	DWFT	685
Hi*erodula timorensis*	COX3	Extracted	DWFT	789
Hi*erodula timorensis*	CYTB	Extracted	DWFT	1137
Hi*erodula timorensis*	ND1	Extracted	DWFT	936
Hi*erodula timorensis*	ND2	Extracted	DWFT	1029
Hi*erodula timorensis*	ND3	Extracted	DWFT	354
Hi*erodula timorensis*	ND4	Extracted	DWFT	1338
Hi*erodula timorensis*	ND4L	Extracted	DWFT	282
Hi*erodula timorensis*	ND5	Extracted	DWFT	1720
Hi*erodula timorensis*	ND6	Extracted	DWFT	504
Hi*erodula zhangi*	12S	Extracted	MZ673800	801
Hi*erodula zhangi*	16S	Extracted	MZ673800	1316
Hi*erodula zhangi*	ATP6	Extracted	MZ673800	678
Hi*erodula zhangi*	ATP8	Extracted	MZ673800	159
Hi*erodula zhangi*	COX1	Extracted	MZ673800	1545
Hi*erodula zhangi*	COX2	Extracted	MZ673800	685
Hi*erodula zhangi*	COX3	Extracted	MZ673800	792
Hi*erodula zhangi*	CYTB	Extracted	MZ673800	1137
Hi*erodula zhangi*	ND1	Extracted	MZ673800	936
Hi*erodula zhangi*	ND2	Extracted	MZ673800	1029
Hi*erodula zhangi*	ND3	Extracted	MZ673800	354
Hi*erodula zhangi*	ND4	Extracted	MZ673800	1338
Hi*erodula zhangi*	ND4L	Extracted	MZ673800	282
Hi*erodula zhangi*	ND5	Extracted	MZ673800	1720
Hi*erodula zhangi*	ND6	Extracted	MZ673800	504
Ephierodula *celebensis*	12S	Extracted	FJ806133	374
Ephierodula *celebensis*	16S	Extracted	FJ806316	518
Ephierodula *celebensis*	ATP6	Missing	NA	0
Ephierodula *celebensis*	ATP8	Missing	NA	0
Ephierodula *celebensis*	COX1	Extracted	FJ802914	1427
Ephierodula *celebensis*	COX2	Missing	NA	0

Species	Gene	Status	Accession	Length(bp)
Ephierodula *celebensis*	COX3	Missing	NA	0
Ephierodula *celebensis*	CYTB	Missing	NA	0
Ephierodula *celebensis*	ND1	Missing	NA	0
Ephierodula *celebensis*	ND2	Missing	NA	0
Ephierodula *celebensis*	ND3	Missing	NA	0
Ephierodula *celebensis*	ND4	Extracted	FJ802739	1225
Ephierodula *celebensis*	ND4L	Missing	NA	0
Ephierodula *celebensis*	ND5	Missing	NA	0
Ephierodula *celebensis*	ND6	Missing	NA	0
Ephierodula *reticulata*	12S	Extracted	FJ806130	67
Ephierodula *reticulata*	16S	Extracted	FJ806313	512
Ephierodula *reticulata*	ATP6	Missing	NA	0
Ephierodula *reticulata*	ATP8	Missing	NA	0
Ephierodula *reticulata*	COX1	Extracted	FJ802911	1425
Ephierodula *reticulata*	COX2	Extracted	FJ807029	670
Ephierodula *reticulata*	COX3	Missing	NA	0
Ephierodula *reticulata*	CYTB	Missing	NA	0
Ephierodula *reticulata*	ND1	Missing	NA	0
Ephierodula *reticulata*	ND2	Missing	NA	0
Ephierodula *reticulata*	ND3	Missing	NA	0
Ephierodula *reticulata*	ND4	Extracted	FJ802736	1268
Ephierodula *reticulata*	ND4L	Missing	NA	0
Ephierodula *reticulata*	ND5	Missing	NA	0
Ephierodula *reticulata*	ND6	Missing	NA	0
Macr*otermes barneyi*	12S	Extracted	JX050221	962
Ma*crotermes barneyi*	16S	Extracted	JX050221	1311
M*acrotermes barneyi*	ATP6	Extracted	JX050221	681
Ma*crotermes barneyi*	ATP8	Extracted	JX050221	162
Ma*crotermes barneyi*	COX1	Extracted	JX050221	1545
M*acrotermes barneyi*	COX2	Extracted	JX050221	679
Ma*crotermes barneyi*	COX3	Extracted	JX050221	789
Ma*crotermes barneyi*	CYTB	Extracted	JX050221	1134
M*acrotermes barneyi*	ND1	Extracted	JX050221	942
Ma*crotermes barneyi*	ND2	Extracted	JX050221	1032
M*acrotermes barneyi*	ND3	Extracted	JX050221	354
Ma*crotermes barneyi*	ND4	Extracted	JX050221	1341
M*acrotermes barneyi*	ND4L	Extracted	JX050221	288
Ma*crotermes barneyi*	ND5	Extracted	JX050221	1720
M*acrotermes barneyi*	ND6	Extracted	JX050221	492
Ma*ntis religiosa*	12S	Extracted	KU201317	793
Ma*ntis religiosa*	16S	Extracted	KU201317	1317
M*antis religiosa*	ATP6	Extracted	KU201317	681
Ma*ntis religiosa*	ATP8	Extracted	KU201317	159

Species	Gene	Status	Accession	Length(bp)
Ma*ntis religiosa*	COX1	Extracted	KU201317	1539
Ma*ntis religiosa*	COX2	Extracted	KU201317	685
Ma*ntis religiosa*	COX3	Extracted	KU201317	792
Ma*ntis religiosa*	CYTB	Extracted	KU201317	1137
Ma*ntis religiosa*	ND1	Extracted	KU201317	936
Ma*ntis religiosa*	ND2	Extracted	KU201317	1027
Ma*ntis religiosa*	ND3	Extracted	KU201317	354
Ma*ntis religiosa*	ND4	Extracted	KU201317	1338
Ma*ntis religiosa*	ND4L	Extracted	KU201317	282
Ma*ntis religiosa*	ND5	Extracted	KU201317	1720
Ma*ntis religiosa*	ND6	Extracted	KU201317	504
Ma*ntoida schraderi*	12S	Extracted	EF383155	376
Ma*ntoida schraderi*	16S	Extracted	EF383315	448
Ma*ntoida schraderi*	ATP6	Missing	NA	0
Mant*oida schraderi*	ATP8	Missing	NA	0
Mant*oida schraderi*	COX1	Extracted	EF383799	1327
M*antoida schraderi*	COX2	Extracted	AY491275	563
Ma*ntoida schraderi*	COX3	Missing	NA	0
Mant*oida schraderi*	CYTB	Missing	NA	0
Mant*oida schraderi*	ND1	Missing	NA	0
Mant*oida schraderi*	ND2	Missing	NA	0
Mant*oida schraderi*	ND3	Missing	NA	0
Mant*oida schraderi*	ND4	Extracted	FJ802424	1278
M*antoida schraderi*	ND4L	Missing	NA	0
Mant*oida schraderi*	ND5	Missing	NA	0
Mant*oida schraderi*	ND6	Missing	NA	0
Mant*oida* sp. MN109	12S	Extracted	FJ806014	370
Ma*ntoida* sp. MN109	16S	Extracted	FJ806189	510
Ma*ntoida* sp. MN109	ATP6	Missing	NA	0
Mant*oida* sp. MN109	ATP8	Missing	NA	0
Mant*oida* sp. MN109	COX1	Extracted	FJ802793	1427
M*antoida* sp. MN109	COX2	Extracted	FJ806903	650
Ma*ntoida* sp. MN109	COX3	Missing	NA	0
Mant*oida* sp. MN109	CYTB	Missing	NA	0
Mant*oida* sp. MN109	ND1	Missing	NA	0
Mant*oida* sp. MN109	ND2	Missing	NA	0
Mant*oida* sp. MN109	ND3	Missing	NA	0
Mant*oida* sp. MN109	ND4	Extracted	FJ802523	1278
M*antoida* sp. MN109	ND4L	Missing	NA	0
Mant*oida* sp. MN109	ND5	Missing	NA	0
Mant*oida* sp. MN109	ND6	Missing	NA	0
Mant*oida* sp. MN110	12S	Extracted	FJ806015	370
Ma*ntoida* sp. MN110	16S	Extracted	FJ806190	515

Species	Gene	Status	Accession	Length(bp)
Ma*ntoida* sp. MN110	ATP6	Missing	NA	0
Mant*oida* sp. MN110	ATP8	Missing	NA	0
Mant*oida* sp. MN110	COX1	Extracted	FJ802794	1427
Ma*ntoida* sp. MN110	COX2	Extracted	FJ806904	670
Ma*ntoida* sp. MN110	COX3	Missing	NA	0
Mant*oida* sp. MN110	CYTB	Missing	NA	0
Mant*oida* sp. MN110	ND1	Missing	NA	0
Mant*oida* sp. MN110	ND2	Missing	NA	0
Mant*oida* sp. MN110	ND3	Missing	NA	0
Mant*oida* sp. MN110	ND4	Extracted	FJ802524	1278
Ma*ntoida* sp. MN110	ND4L	Missing	NA	0
Mant*oida* sp. MN110	ND5	Missing	NA	0
Mant*oida* sp. MN110	ND6	Missing	NA	0
Mant*oida* sp. MN179	12S	Extracted	EF383249	377
Ma*ntoida* sp. MN179	16S	Extracted	EF383408	555
Ma*ntoida* sp. MN179	ATP6	Missing	NA	0
Mant*oida* sp. MN179	ATP8	Missing	NA	0
Mant*oida* sp. MN179	COX1	Extracted	EF383890	1414
Ma*ntoida* sp. MN179	COX2	Extracted	EF384019	636
Ma*ntoida* sp. MN179	COX3	Missing	NA	0
Mant*oida* sp. MN179	CYTB	Missing	NA	0
Mant*oida* sp. MN179	ND1	Missing	NA	0
Mant*oida* sp. MN179	ND2	Missing	NA	0
Mant*oida* sp. MN179	ND3	Missing	NA	0
Mant*oida* sp. MN179	ND4	Extracted	FJ802585	1278
Ma*ntoida* sp. MN179	ND4L	Missing	NA	0
Mant*oida* sp. MN179	ND5	Missing	NA	0
Mant*oida* sp. MN179	ND6	Missing	NA	0
Mant*oida* sp. MN180	12S	Extracted	FJ806044	371
Ma*ntoida* sp. MN180	16S	Extracted	FJ806220	442
Ma*ntoida* sp. MN180	ATP6	Missing	NA	0
Mant*oida* sp. MN180	ATP8	Missing	NA	0
Mant*oida* sp. MN180	COX1	Extracted	FJ802822	1427
Ma*ntoida* sp. MN180	COX2	Extracted	FJ806935	670
Ma*ntoida* sp. MN180	COX3	Missing	NA	0
Mant*oida* sp. MN180	CYTB	Missing	NA	0
Mant*oida* sp. MN180	ND1	Missing	NA	0
Mant*oida* sp. MN180	ND2	Missing	NA	0
Mant*oida* sp. MN180	ND3	Missing	NA	0
Mant*oida* sp. MN180	ND4	Extracted	FJ802586	1278
Ma*ntoida* sp. MN180	ND4L	Missing	NA	0
Mant*oida* sp. MN180	ND5	Missing	NA	0
Mant*oida* sp. MN180	ND6	Missing	NA	0

Species	Gene	Status	Accession	Length(bp)
Mastotermes darwiniensis	12S	Extracted	OM646554	819
Mastotermes darwiniensis	16S	Extracted	OM646554	1301
Mastotermes darwiniensis	ATP6	Extracted	OM646554	681
Mastotermes darwiniensis	ATP8	Extracted	OM646554	159
Mastotermes darwiniensis	COX1	Extracted	OM646554	1542
Mastotermes darwiniensis	COX2	Extracted	OM646554	687
Mastotermes darwiniensis	COX3	Extracted	OM646554	789
Mastotermes darwiniensis	CYTB	Extracted	OM646554	1134
Mastotermes darwiniensis	ND1	Extracted	OM646554	945
Mastotermes darwiniensis	ND2	Extracted	OM646554	1029
Mastotermes darwiniensis	ND3	Extracted	OM646554	354
Mastotermes darwiniensis	ND4	Extracted	OM646554	1341
Mastotermes darwiniensis	ND4L	Extracted	OM646554	288
Mastotermes darwiniensis	ND5	Extracted	OM646554	1729
Mastotermes darwiniensis	ND6	Extracted	OM646554	495
Mesopteryx alata	12S	Extracted	KX434813	780
Mesopteryx alata	16S	Extracted	KX434813	1309
Mesopteryx alata	ATP6	Extracted	KX434813	678
Mesopteryx alata	ATP8	Extracted	KX434813	159
Mesopteryx alata	COX1	Extracted	KX434813	1541
Mesopteryx alata	COX2	Extracted	KX434813	685
Mesopteryx alata	COX3	Extracted	KX434813	792
Mesopteryx alata	CYTB	Extracted	KX434813	1137
Mesopteryx alata	ND1	Extracted	KX434813	936
Mesopteryx alata	ND2	Extracted	KX434813	1029
Mesopteryx alata	ND3	Extracted	KX434813	354
Mesopteryx alata	ND4	Extracted	KX434813	1338
Mesopteryx alata	ND4L	Extracted	KX434813	282
Mesopteryx alata	ND5	Extracted	KX434813	1723
Mesopteryx alata	ND6	Extracted	KX434813	504
Neodanuria bolauana	12S	Extracted	KX434858	781
Neodanuria bolauana	16S	Extracted	KX434858	1308
Neodanuria bolauana	ATP6	Extracted	KX434858	678
Neodanuria bolauana	ATP8	Extracted	KX434858	159
Neodanuria bolauana	COX1	Extracted	KX434858	1539
Neodanuria bolauana	COX2	Extracted	KX434858	685
Neodanuria bolauana	COX3	Extracted	KX434858	789
Neodanuria bolauana	CYTB	Extracted	KX434858	1140
Neodanuria bolauana	ND1	Extracted	KX434858	915
Neodanuria bolauana	ND2	Extracted	KX434858	1026
Neodanuria bolauana	ND3	Extracted	KX434858	354
Neodanuria bolauana	ND4	Extracted	KX434858	1338
Neodanuria bolauana	ND4L	Extracted	KX434858	282

Species	Gene	Status	Accession	Length(bp)
Neodanuria bolauana	ND5	Extracted	KX434858	1720
Neodanuria bolauana	ND6	Extracted	KX434858	504
Omomantis sp. MN276	12S	Extracted	EF383304	373
Omomantis sp. MN276	16S	Extracted	EF383463	556
Omomantis sp. MN276	ATP6	Missing	NA	0
Omomantis sp. MN276	ATP8	Missing	NA	0
Omomantis sp. MN276	COX1	Extracted	EF383944	1414
Omomantis sp. MN276	COX2	Extracted	EF384075	636
Omomantis sp. MN276	COX3	Missing	NA	0
Omomantis sp. MN276	CYTB	Missing	NA	0
Omomantis sp. MN276	ND1	Missing	NA	0
Omomantis sp. MN276	ND2	Missing	NA	0
Omomantis sp. MN276	ND3	Missing	NA	0
Omomantis sp. MN276	ND4	Extracted	FJ802671	1278
Omomantis sp. MN276	ND4L	Missing	NA	0
Omomantis sp. MN276	ND5	Missing	NA	0
Omomantis sp. MN276	ND6	Missing	NA	0
Orthodera ministralis	12S	Extracted	KX434842	773
Orthodera ministralis	16S	Extracted	KX434842	1310
Orthodera ministralis	ATP6	Extracted	KX434842	678
Orthodera ministralis	ATP8	Extracted	KX434842	159
Orthodera ministralis	COX1	Extracted	KX434842	1536
Orthodera ministralis	COX2	Extracted	KX434842	685
Orthodera ministralis	COX3	Extracted	KX434842	792
Orthodera ministralis	CYTB	Extracted	KX434842	1140
Orthodera ministralis	ND1	Extracted	KX434842	936
Orthodera ministralis	ND2	Extracted	KX434842	1029
Orthodera ministralis	ND3	Extracted	KX434842	354
Orthodera ministralis	ND4	Extracted	KX434842	1338
Orthodera ministralis	ND4L	Extracted	KX434842	282
Orthodera ministralis	ND5	Extracted	KX434842	1720
Orthodera ministralis	ND6	Extracted	KX434842	504
Orthodera novaezealandiae	12S	Extracted	BBYOY	772
Orthodera novaezealandiae	16S	Extracted	BBYOY	1312
Orthodera novaezealandiae	ATP6	Extracted	BBYOY	678
Orthodera novaezealandiae	ATP8	Extracted	BBYOY	159
Orthodera novaezealandiae	COX1	Extracted	BBYOY	1536
Orthodera novaezealandiae	COX2	Extracted	BBYOY	685
Orthodera novaezealandiae	COX3	Extracted	BBYOY	789
Orthodera novaezealandiae	CYTB	Extracted	BBYOY	1140
Orthodera novaezealandiae	ND1	Extracted	BBYOY	936
Orthodera novaezealandiae	ND2	Extracted	BBYOY	1029
Orthodera novaezealandiae	ND3	Extracted	BBYOY	354

Species	Gene	Status	Accession	Length(bp)
Orthodera novaezealandiae	ND4	Extracted	BBYOY	1338
Orthodera novaezealandiae	ND4L	Extracted	BBYOY	282
Orthodera novaezealandiae	ND5	Extracted	BBYOY	1720
Orthodera novaezealandiae	ND6	Extracted	BBYOY	504
Orthodera sp. MN033	12S	Extracted	FJ805987	372
Orthodera sp. MN033	16S	Extracted	FJ806163	515
Orthodera sp. MN033	ATP6	Missing	NA	0
Orthodera sp. MN033	ATP8	Missing	NA	0
Orthodera sp. MN033	COX1	Extracted	FJ802766	1354
Orthodera sp. MN033	COX2	Extracted	AY491299	563
Orthodera sp. MN033	COX3	Missing	NA	0
Orthodera sp. MN033	CYTB	Missing	NA	0
Orthodera sp. MN033	ND1	Missing	NA	0
Orthodera sp. MN033	ND2	Missing	NA	0
Orthodera sp. MN033	ND3	Missing	NA	0
Orthodera sp. MN033	ND4	Extracted	FJ802448	1278
Orthodera sp. MN033	ND4L	Missing	NA	0
Orthodera sp. MN033	ND5	Missing	NA	0
Orthodera sp. MN033	ND6	Missing	NA	0
Oxyopsis peruviana	12S	Extracted	NMLT	605
Oxyopsis peruviana	16S	Extracted	NMLT	1329
Oxyopsis peruviana	ATP6	Extracted	NMLT	681
Oxyopsis peruviana	ATP8	Extracted	NMLT	159
Oxyopsis peruviana	COX1	Extracted	NMLT	1542
Oxyopsis peruviana	COX2	Extracted	NMLT	685
Oxyopsis peruviana	COX3	Extracted	NMLT	792
Oxyopsis peruviana	CYTB	Extracted	NMLT	1137
Oxyopsis peruviana	ND1	Extracted	NMLT	936
Oxyopsis peruviana	ND2	Extracted	NMLT	1011
Oxyopsis peruviana	ND3	Extracted	NMLT	354
Oxyopsis peruviana	ND4	Extracted	NMLT	1338
Oxyopsis peruviana	ND4L	Extracted	NMLT	282
Oxyopsis peruviana	ND5	Extracted	NMLT	1723
Oxyopsis peruviana	ND6	Extracted	NMLT	504
Oxyopsis sp. MN294	12S	Extracted	FJ806094	372
Oxyopsis sp. MN294	16S	Extracted	FJ806267	521
Oxyopsis sp. MN294	ATP6	Missing	NA	0
Oxyopsis sp. MN294	ATP8	Missing	NA	0
Oxyopsis sp. MN294	COX1	Extracted	FJ802868	694
Oxyopsis sp. MN294	COX2	Extracted	FJ806983	670
Oxyopsis sp. MN294	COX3	Missing	NA	0
Oxyopsis sp. MN294	CYTB	Missing	NA	0
Oxyopsis sp. MN294	ND1	Missing	NA	0

Species	Gene	Status	Accession	Length(bp)
Oxyo*psis* sp. MN294	ND2	Missing	NA	0
Oxyo*psis* sp. MN294	ND3	Missing	NA	0
Oxyo*psis* sp. MN294	ND4	Extracted	FJ802689	1278
O*xyopsis* sp. MN294	ND4L	Missing	NA	0
Oxyo*psis* sp. MN294	ND5	Missing	NA	0
Oxyo*psis* sp. MN294	ND6	Missing	NA	0
Para*stagmatoptera* sp. MN028	12S	Extracted	FJ805983	374
P*arastagmatoptera* sp. MN028	16S	Extracted	FJ806159	514
P*arastagmatoptera* sp. MN028	ATP6	Missing	NA	0
Para*stagmatoptera* sp. MN028	ATP8	Missing	NA	0
Para*stagmatoptera* sp. MN028	COX1	Extracted	FJ802762	1376
P*arastagmatoptera* sp. MN028	COX2	Extracted	AY491294	563
P*arastagmatoptera* sp. MN028	COX3	Missing	NA	0
Para*stagmatoptera* sp. MN028	CYTB	Missing	NA	0
Para*stagmatoptera* sp. MN028	ND1	Missing	NA	0
Para*stagmatoptera* sp. MN028	ND2	Missing	NA	0
Para*stagmatoptera* sp. MN028	ND3	Missing	NA	0
Para*stagmatoptera* sp. MN028	ND4	Extracted	FJ802443	1278
P*arastagmatoptera* sp. MN028	ND4L	Missing	NA	0
Para*stagmatoptera* sp. MN028	ND5	Missing	NA	0
Para*stagmatoptera* sp. MN028	ND6	Missing	NA	0
Peri*planeta americana*	12S	Extracted	NC 016956	805
P*eriplaneta americana*	16S	Extracted	NC 016956	1292
P*eriplaneta americana*	ATP6	Extracted	NC 016956	681
P*eriplaneta americana*	ATP8	Extracted	NC 016956	159
P*eriplaneta americana*	COX1	Extracted	NC 016956	1539
P*eriplaneta americana*	COX2	Extracted	NC 016956	685
P*eriplaneta americana*	COX3	Extracted	NC 016956	789
P*eriplaneta americana*	CYTB	Extracted	NC 016956	1134
P*eriplaneta americana*	ND1	Extracted	NC 016956	948
P*eriplaneta americana*	ND2	Extracted	NC 016956	1026
P*eriplaneta americana*	ND3	Extracted	NC 016956	354
P*eriplaneta americana*	ND4	Extracted	NC 016956	1338
P*eriplaneta americana*	ND4L	Extracted	NC 016956	285
P*eriplaneta americana*	ND5	Extracted	NC 016956	1731
P*eriplaneta americana*	ND6	Extracted	NC 016956	501
Ph*asmomantella sp.*	12S	Extracted	YNXHT01	595
Phasmomantella sp.	16S	Extracted	YNXHT01	1313
Phasmomantella sp.	ATP6	Extracted	YNXHT01	678
Phasmomantella sp.	ATP8	Extracted	YNXHT01	159
Phasmomantella sp.	COX1	Extracted	YNXHT01	1536
Phasmomantella sp.	COX2	Extracted	YNXHT01	691
Phasmomantella sp.	COX3	Extracted	YNXHT01	789

Species	Gene	Status	Accession	Length(bp)
Phasmomantella sp.	CYTB	Extracted	YNXHT01	1137
Phasmomantella sp.	ND1	Extracted	YNXHT01	933
Phasmomantella sp.	ND2	Extracted	YNXHT01	1026
Phasmomantella sp.	ND3	Extracted	YNXHT01	354
Phasmomantella sp.	ND4	Extracted	YNXHT01	1338
Phasmomantella sp.	ND4L	Extracted	YNXHT01	282
Phasmomantella sp.	ND5	Extracted	YNXHT01	1726
Phasmomantella sp.	ND6	Extracted	YNXHT01	504
Phasmomantis	12S	Extracted	MXGJR	788
Phasmomantis	16S	Extracted	MXGJR	1319
Phasmomantis	ATP6	Extracted	MXGJR	678
Phasmomantis	ATP8	Extracted	MXGJR	159
Phasmomantis	COX1	Extracted	MXGJR	1542
Phasmomantis	COX2	Extracted	MXGJR	685
Phasmomantis	COX3	Extracted	MXGJR	789
Phasmomantis	CYTB	Extracted	MXGJR	1134
Phasmomantis	ND1	Extracted	MXGJR	936
Phasmomantis	ND2	Extracted	MXGJR	1032
Phasmomantis	ND3	Extracted	MXGJR	354
Phasmomantis	ND4	Extracted	MXGJR	1338
Phasmomantis	ND4L	Extracted	MXGJR	282
Phasmomantis	ND5	Extracted	MXGJR	1723
Phasmomantis	ND6	Extracted	MXGJR	504
Plistospilota sp. JZ-2017	12S	Extracted	KX434821	795
Plistospilota sp. JZ-2017	16S	Extracted	KX434821	1320
Plistospilota sp. JZ-2017	ATP6	Extracted	KX434821	678
Plistospilota sp. JZ-2017	ATP8	Extracted	KX434821	159
Plistospilota sp. JZ-2017	COX1	Extracted	KX434821	1542
Plistospilota sp. JZ-2017	COX2	Extracted	KX434821	685
Plistospilota sp. JZ-2017	COX3	Extracted	KX434821	789
Plistospilota sp. JZ-2017	CYTB	Extracted	KX434821	1137
Plistospilota sp. JZ-2017	ND1	Extracted	KX434821	936
Plistospilota sp. JZ-2017	ND2	Extracted	KX434821	1029
Plistospilota sp. JZ-2017	ND3	Extracted	KX434821	354
Plistospilota sp. JZ-2017	ND4	Extracted	KX434821	1326
Plistospilota sp. JZ-2017	ND4L	Extracted	KX434821	282
Plistospilota sp. JZ-2017	ND5	Extracted	KX434821	1723
Plistospilota sp. JZ-2017	ND6	Extracted	KX434821	501
Pnigomantis medioconstricta	12S	Extracted	KX434851	778
Pnigomantis medioconstricta	16S	Extracted	KX434851	1312
Pnigomantis medioconstricta	ATP6	Extracted	KX434851	678
Pnigomantis medioconstricta	ATP8	Extracted	KX434851	159
Pnigomantis medioconstricta	COX1	Extracted	KX434851	1536

Species	Gene	Status	Accession	Length(bp)
P*nigomantis medioconstricta*	COX2	Extracted	KX434851	685
P*nigomantis medioconstricta*	COX3	Extracted	KX434851	792
P*nigomantis medioconstricta*	CYTB	Extracted	KX434851	1137
P*nigomantis medioconstricta*	ND1	Extracted	KX434851	936
P*nigomantis medioconstricta*	ND2	Extracted	KX434851	1029
P*nigomantis medioconstricta*	ND3	Extracted	KX434851	354
P*nigomantis medioconstricta*	ND4	Extracted	KX434851	1338
P*nigomantis medioconstricta*	ND4L	Extracted	KX434851	282
P*nigomantis medioconstricta*	ND5	Extracted	KX434851	1723
P*nigomantis medioconstricta*	ND6	Extracted	KX434851	504
Po*lyspilota aeruginosa*	12S	Extracted	FJ806068	371
Po*lyspilota aeruginosa*	16S	Extracted	FJ806244	517
Po*lyspilota aeruginosa*	ATP6	Missing	NA	0
Poly*spilota aeruginosa*	ATP8	Missing	NA	0
Poly*spilota aeruginosa*	COX1	Extracted	FJ802847	1427
Po*lyspilota aeruginosa*	COX2	Extracted	FJ806958	670
Po*lyspilota aeruginosa*	COX3	Missing	NA	0
Poly*spilota aeruginosa*	CYTB	Missing	NA	0
Poly*spilota aeruginosa*	ND1	Missing	NA	0
Poly*spilota aeruginosa*	ND2	Missing	NA	0
Poly*spilota aeruginosa*	ND3	Missing	NA	0
Poly*spilota aeruginosa*	ND4	Extracted	FJ802647	1278
Po*lyspilota aeruginosa*	ND4L	Missing	NA	0
Poly*spilota aeruginosa*	ND5	Missing	NA	0
Poly*spilota aeruginosa*	ND6	Missing	NA	0
Popa *spurca*	12S	Extracted	EF383227	381
Po*pa spurca*	16S	Extracted	EF383387	515
Po*pa spurca*	ATP6	Missing	NA	0
Popa *spurca*	ATP8	Missing	NA	0
Popa *spurca*	COX1	Extracted	EF383870	1414
Po*pa spurca*	COX2	Extracted	EF383997	631
Po*pa spurca*	COX3	Missing	NA	0
Popa *spurca*	CYTB	Missing	NA	0
Popa *spurca*	ND1	Missing	NA	0
Popa *spurca*	ND2	Missing	NA	0
Popa *spurca*	ND3	Missing	NA	0
Popa *spurca*	ND4	Extracted	FJ802546	1278
P*opa spurca*	ND4L	Missing	NA	0
Popa *spurca*	ND5	Missing	NA	0
Popa *spurca*	ND6	Missing	NA	0
Pro*hierodula ornatipennis*	12S	Extracted	EF383291	379
Pr*ohierodula ornatipennis*	16S	Extracted	EF383449	562
Pr*ohierodula ornatipennis*	ATP6	Missing	NA	0

Species	Gene	Status	Accession	Length(bp)
Prohierodula ornatipennis	ATP8	Missing	NA	0
Prohierodula ornatipennis	COX1	Extracted	EF383930	1414
Prohierodula ornatipennis	COX2	Extracted	EF384060	636
Prohierodula ornatipennis	COX3	Missing	NA	0
Prohierodula ornatipennis	CYTB	Missing	NA	0
Prohierodula ornatipennis	ND1	Missing	NA	0
Prohierodula ornatipennis	ND2	Missing	NA	0
Prohierodula ornatipennis	ND3	Missing	NA	0
Prohierodula ornatipennis	ND4	Extracted	FJ802648	1278
Prohierodula ornatipennis	ND4L	Missing	NA	0
Prohierodula ornatipennis	ND5	Missing	NA	0
Prohierodula ornatipennis	ND6	Missing	NA	0
Prohierodula picta	12S	Extracted	KX434855	799
Prohierodula picta	16S	Extracted	KX434855	1323
Prohierodula picta	ATP6	Extracted	KX434855	678
Prohierodula picta	ATP8	Extracted	KX434855	159
Prohierodula picta	COX1	Extracted	KX434855	1557
Prohierodula picta	COX2	Extracted	KX434855	685
Prohierodula picta	COX3	Extracted	KX434855	792
Prohierodula picta	CYTB	Extracted	KX434855	1137
Prohierodula picta	ND1	Extracted	KX434855	933
Prohierodula picta	ND2	Extracted	KX434855	1029
Prohierodula picta	ND3	Extracted	KX434855	354
Prohierodula picta	ND4	Extracted	KX434855	1338
Prohierodula picta	ND4L	Extracted	KX434855	282
Prohierodula picta	ND5	Extracted	KX434855	1723
Prohierodula picta	ND6	Extracted	KX434855	504
Pseudempusa pinnapavonis	12S	Extracted	OM202557	814
Pseudempusa pinnapavonis	16S	Extracted	OM202557	1303
Pseudempusa pinnapavonis	ATP6	Extracted	OM202557	684
Pseudempusa pinnapavonis	ATP8	Extracted	OM202557	159
Pseudempusa pinnapavonis	COX1	Extracted	OM202557	1542
Pseudempusa pinnapavonis	COX2	Extracted	OM202557	685
Pseudempusa pinnapavonis	COX3	Extracted	OM202557	791
Pseudempusa pinnapavonis	CYTB	Extracted	OM202557	1137
Pseudempusa pinnapavonis	ND1	Extracted	OM202557	936
Pseudempusa pinnapavonis	ND2	Extracted	OM202557	1023
Pseudempusa pinnapavonis	ND3	Extracted	OM202557	354
Pseudempusa pinnapavonis	ND4	Extracted	OM202557	1336
Pseudempusa pinnapavonis	ND4L	Extracted	OM202557	282
Pseudempusa pinnapavonis	ND5	Extracted	OM202557	1723
Pseudempusa pinnapavonis	ND6	Extracted	OM202557	501
Pseudomantis albofimbriata	12S	Extracted	BXNT	775

Species	Gene	Status	Accession	Length(bp)
P*seudomantis albofimbriata*	16S	Extracted	BXNT	1320
P*seudomantis albofimbriata*	ATP6	Extracted	BXNT	678
P*seudomantis albofimbriata*	ATP8	Extracted	BXNT	159
P*seudomantis albofimbriata*	COX1	Extracted	BXNT	1536
P*seudomantis albofimbriata*	COX2	Extracted	BXNT	685
P*seudomantis albofimbriata*	COX3	Extracted	BXNT	789
P*seudomantis albofimbriata*	CYTB	Extracted	BXNT	1137
P*seudomantis albofimbriata*	ND1	Extracted	BXNT	936
P*seudomantis albofimbriata*	ND2	Extracted	BXNT	1029
P*seudomantis albofimbriata*	ND3	Extracted	BXNT	354
P*seudomantis albofimbriata*	ND4	Extracted	BXNT	1338
P*seudomantis albofimbriata*	ND4L	Extracted	BXNT	282
P*seudomantis albofimbriata*	ND5	Extracted	BXNT	1720
P*seudomantis albofimbriata*	ND6	Extracted	BXNT	504
P*seudovates chlorophaea*	12S	Extracted	KX434838	608
P*seudovates chlorophaea*	16S	Extracted	KX434838	1316
P*seudovates chlorophaea*	ATP6	Extracted	KX434838	678
P*seudovates chlorophaea*	ATP8	Extracted	KX434838	159
P*seudovates chlorophaea*	COX1	Extracted	KX434838	1536
P*seudovates chlorophaea*	COX2	Extracted	KX434838	685
P*seudovates chlorophaea*	COX3	Extracted	KX434838	792
P*seudovates chlorophaea*	CYTB	Extracted	KX434838	1137
P*seudovates chlorophaea*	ND1	Extracted	KX434838	927
P*seudovates chlorophaea*	ND2	Extracted	KX434838	1027
P*seudovates chlorophaea*	ND3	Extracted	KX434838	354
P*seudovates chlorophaea*	ND4	Extracted	KX434838	1339
P*seudovates chlorophaea*	ND4L	Extracted	KX434838	282
P*seudovates chlorophaea*	ND5	Extracted	KX434838	1723
P*seudovates chlorophaea*	ND6	Extracted	KX434838	504
P*seudovates cingulata*	12S	Extracted	FJ806060	371
P*seudovates cingulata*	16S	Extracted	FJ806236	513
P*seudovates cingulata*	ATP6	Missing	NA	0
Pseu*dovates cingulata*	ATP8	Missing	NA	0
Pseu*dovates cingulata*	COX1	Extracted	FJ802839	1427
P*seudovates cingulata*	COX2	Extracted	FJ806951	670
P*seudovates cingulata*	COX3	Missing	NA	0
Pseu*dovates cingulata*	CYTB	Missing	NA	0
Pseu*dovates cingulata*	ND1	Missing	NA	0
Pseu*dovates cingulata*	ND2	Missing	NA	0
Pseu*dovates cingulata*	ND3	Missing	NA	0
Pseu*dovates cingulata*	ND4	Extracted	FJ802619	1278
P*seudovates cingulata*	ND4L	Missing	NA	0
Pseu*dovates cingulata*	ND5	Missing	NA	0

Species	Gene	Status	Accession	Length(bp)
Pseudovates cingulata	ND6	Missing	NA	0
Pseudovates denticulata	12S	Missing	NA	0
Pseudovates denticulata	16S	Extracted	FJ806283	512
Pseudovates denticulata	ATP6	Missing	NA	0
Pseudovates denticulata	ATP8	Missing	NA	0
Pseudovates denticulata	COX1	Extracted	FJ802882	647
Pseudovates denticulata	COX2	Extracted	FJ807000	670
Pseudovates denticulata	COX3	Missing	NA	0
Pseudovates denticulata	CYTB	Missing	NA	0
Pseudovates denticulata	ND1	Missing	NA	0
Pseudovates denticulata	ND2	Missing	NA	0
Pseudovates denticulata	ND3	Missing	NA	0
Pseudovates denticulata	ND4	Extracted	FJ802706	1278
Pseudovates denticulata	ND4L	Missing	NA	0
Pseudovates denticulata	ND5	Missing	NA	0
Pseudovates denticulata	ND6	Missing	NA	0
Pseudovates peruviana	12S	Extracted	MW357301	770
Pseudovates peruviana	16S	Extracted	MW357301	1313
Pseudovates peruviana	ATP6	Extracted	MW357301	678
Pseudovates peruviana	ATP8	Extracted	MW357301	159
Pseudovates peruviana	COX1	Extracted	MW357301	1536
Pseudovates peruviana	COX2	Extracted	MW357301	685
Pseudovates peruviana	COX3	Extracted	MW357301	792
Pseudovates peruviana	CYTB	Extracted	MW357301	1134
Pseudovates peruviana	ND1	Extracted	MW357301	936
Pseudovates peruviana	ND2	Extracted	MW357301	1032
Pseudovates peruviana	ND3	Extracted	MW357301	354
Pseudovates peruviana	ND4	Extracted	MW357301	1338
Pseudovates peruviana	ND4L	Extracted	MW357301	282
Pseudovates peruviana	ND5	Extracted	MW357301	1720
Pseudovates peruviana	ND6	Extracted	MW357301	504
Rhombodera basalis	12S	Extracted	173MLCYJRDTqd	803
Rhombodera basalis	16S	Extracted	173MLCYJRDTqd	1312
Rhombodera basalis	ATP6	Extracted	173MLCYJRDTqd	678
Rhombodera basalis	ATP8	Extracted	173MLCYJRDTqd	159
Rhombodera basalis	COX1	Extracted	173MLCYJRDTqd	1542
Rhombodera basalis	COX2	Extracted	173MLCYJRDTqd	685
Rhombodera basalis	COX3	Extracted	173MLCYJRDTqd	792
Rhombodera basalis	CYTB	Extracted	173MLCYJRDTqd	1137
Rhombodera basalis	ND1	Extracted	173MLCYJRDTqd	942
Rhombodera basalis	ND2	Extracted	173MLCYJRDTqd	1029
Rhombodera basalis	ND3	Extracted	173MLCYJRDTqd	354
Rhombodera basalis	ND4	Extracted	173MLCYJRDTqd	1338

Species	Gene	Status	Accession	Length(bp)
R*hombodera basalis*	ND4L	Extracted	173MLCYJRDTqd	282
Rh*ombodera basalis*	ND5	Extracted	173MLCYJRDTqd	1720
R*hombodera basalis*	ND6	Extracted	173MLCYJRDTqd	504
Rh*ombodera brachynota*	12S	Extracted	KX611802	790
Rh*ombodera brachynota*	16S	Extracted	KX611802	1314
R*hombodera brachynota*	ATP6	Extracted	KX611802	681
Rh*ombodera brachynota*	ATP8	Extracted	KX611802	159
Rh*ombodera brachynota*	COX1	Extracted	KX611802	1536
R*hombodera brachynota*	COX2	Extracted	KX611802	685
Rh*ombodera brachynota*	COX3	Extracted	KX611802	789
Rh*ombodera brachynota*	CYTB	Extracted	KX611802	1137
R*hombodera brachynota*	ND1	Extracted	KX611802	936
Rh*ombodera brachynota*	ND2	Extracted	KX611802	1029
R*hombodera brachynota*	ND3	Extracted	KX611802	354
Rh*ombodera brachynota*	ND4	Extracted	KX611802	1338
R*hombodera brachynota*	ND4L	Extracted	KX611802	282
Rh*ombodera brachynota*	ND5	Extracted	KX611802	1721
R*hombodera brachynota*	ND6	Extracted	KX611802	504
Rh*ombodera stalii*	12S	Extracted	EF383162	381
Rh*ombodera stalii*	16S	Extracted	EF383322	240
Rh*ombodera stalii*	ATP6	Missing	NA	0
Rhom*bodera stalii*	ATP8	Missing	NA	0
Rhom*bodera stalii*	COX1	Extracted	EF383806	1339
R*hombodera stalii*	COX2	Extracted	AY491284	530
Rh*ombodera stalii*	COX3	Missing	NA	0
Rhom*bodera stalii*	CYTB	Missing	NA	0
Rhom*bodera stalii*	ND1	Missing	NA	0
Rhom*bodera stalii*	ND2	Missing	NA	0
Rhom*bodera stalii*	ND3	Missing	NA	0
Rhom*bodera stalii*	ND4	Extracted	FJ802433	1278
R*hombodera stalii*	ND4L	Missing	NA	0
Rhom*bodera stalii*	ND5	Missing	NA	0
Rhom*bodera stalii*	ND6	Missing	NA	0
Rhom*bodera valida*	12S	Extracted	KX611804	794
Rh*ombodera valida*	16S	Extracted	KX611804	1317
R*hombodera valida*	ATP6	Extracted	KX611804	681
Rh*ombodera valida*	ATP8	Extracted	KX611804	159
Rh*ombodera valida*	COX1	Extracted	KX611804	1536
R*hombodera valida*	COX2	Extracted	KX611804	685
Rh*ombodera valida*	COX3	Extracted	KX611804	789
Rh*ombodera valida*	CYTB	Extracted	KX611804	1134
R*hombodera valida*	ND1	Extracted	KX611804	936
Rh*ombodera valida*	ND2	Extracted	KX611804	1029

Species	Gene	Status	Accession	Length(bp)
R*hombodera valida*	ND3	Extracted	KX611804	354
Rh*ombodera valida*	ND4	Extracted	KX611804	1338
R*hombodera valida*	ND4L	Extracted	KX611804	282
Rh*ombodera valida*	ND5	Extracted	KX611804	1724
R*hombodera valida*	ND6	Extracted	KX611804	504
Sp*hodromantis lineola*	12S	Extracted	KY689123	770
Sp*hodromantis lineola*	16S	Extracted	KY689123	1321
Sp*hodromantis lineola*	ATP6	Extracted	KY689123	678
Sp*hodromantis lineola*	ATP8	Extracted	KY689123	159
Sp*hodromantis lineola*	COX1	Extracted	KY689123	1533
Sp*hodromantis lineola*	COX2	Extracted	KY689123	685
Sp*hodromantis lineola*	COX3	Extracted	KY689123	792
Sp*hodromantis lineola*	CYTB	Extracted	KY689123	1137
Sp*hodromantis lineola*	ND1	Extracted	KY689123	936
Sp*hodromantis lineola*	ND2	Extracted	KY689123	1029
Sp*hodromantis lineola*	ND3	Extracted	KY689123	354
Sp*hodromantis lineola*	ND4	Extracted	KY689123	1338
Sp*hodromantis lineola*	ND4L	Extracted	KY689123	282
Sp*hodromantis lineola*	ND5	Extracted	KY689123	1720
Sp*hodromantis lineola*	ND6	Extracted	KY689123	504
Sp*hodromantis viridis*	12S	Extracted	KX434840	778
Sp*hodromantis viridis*	16S	Extracted	KX434840	1314
Sp*hodromantis viridis*	ATP6	Extracted	KX434840	678
Sp*hodromantis viridis*	ATP8	Extracted	KX434840	159
Sp*hodromantis viridis*	COX1	Extracted	KX434840	1539
Sp*hodromantis viridis*	COX2	Extracted	KX434840	685
Sp*hodromantis viridis*	COX3	Extracted	KX434840	792
Sp*hodromantis viridis*	CYTB	Extracted	KX434840	1137
Sp*hodromantis viridis*	ND1	Extracted	KX434840	939
Sp*hodromantis viridis*	ND2	Extracted	KX434840	1029
Sp*hodromantis viridis*	ND3	Extracted	KX434840	354
Sp*hodromantis viridis*	ND4	Extracted	KX434840	1338
Sp*hodromantis viridis*	ND4L	Extracted	KX434840	282
Sp*hodromantis viridis*	ND5	Extracted	KX434840	1723
Sp*hodromantis viridis*	ND6	Extracted	KX434840	504
Sp*hodropoda* sp. MN275	12S	Extracted	EF383303	306
Sp*hodropoda* sp. MN275	16S	Extracted	EF383462	568
Sp*hodropoda* sp. MN275	ATP6	Missing	NA	0
Spho*dropoda* sp. MN275	ATP8	Missing	NA	0
Spho*dropoda* sp. MN275	COX1	Extracted	EF383943	1414
Sp*hodropoda* sp. MN275	COX2	Extracted	EF384074	636
Sp*hodropoda* sp. MN275	COX3	Missing	NA	0
Spho*dropoda* sp. MN275	CYTB	Missing	NA	0

Species	Gene	Status	Accession	Length(bp)
Spho*dropoda* sp. MN275	ND1	Missing	NA	0
Spho*dropoda* sp. MN275	ND2	Missing	NA	0
Spho*dropoda* sp. MN275	ND3	Missing	NA	0
Spho*dropoda* sp. MN275	ND4	Extracted	FJ802670	1278
S*phodropoda* sp. MN275	ND4L	Missing	NA	0
Spho*dropoda* sp. MN275	ND5	Missing	NA	0
Spho*dropoda* sp. MN275	ND6	Missing	NA	0
Spho*dropoda sp.*	12S	Extracted	OZAH	788
Sp*hodropoda sp.*	16S	Extracted	OZAH	1332
S*phodropoda sp.*	ATP6	Extracted	OZAH	681
Sp*hodropoda sp.*	ATP8	Extracted	OZAH	159
Sp*hodropoda sp.*	COX1	Extracted	OZAH	1542
S*phodropoda sp.*	COX2	Extracted	OZAH	685
Sp*hodropoda sp.*	COX3	Extracted	OZAH	789
Sp*hodropoda sp.*	CYTB	Extracted	OZAH	1137
S*phodropoda sp.*	ND1	Extracted	OZAH	936
Sp*hodropoda sp.*	ND2	Extracted	OZAH	1029
S*phodropoda sp.*	ND3	Extracted	OZAH	354
Sp*hodropoda sp.*	ND4	Extracted	OZAH	1338
S*phodropoda sp.*	ND4L	Extracted	OZAH	282
Sp*hodropoda sp.*	ND5	Extracted	OZAH	1724
S*phodropoda sp.*	ND6	Extracted	OZAH	504
St*agmatoptera biocellata*	12S	Extracted	MW357302	769
St*agmatoptera biocellata*	16S	Extracted	MW357302	1309
St*agmatoptera biocellata*	ATP6	Extracted	MW357302	678
St*agmatoptera biocellata*	ATP8	Extracted	MW357302	159
St*agmatoptera biocellata*	COX1	Extracted	MW357302	1536
St*agmatoptera biocellata*	COX2	Extracted	MW357302	685
St*agmatoptera biocellata*	COX3	Extracted	MW357302	792
St*agmatoptera biocellata*	CYTB	Extracted	MW357302	1137
St*agmatoptera biocellata*	ND1	Extracted	MW357302	936
St*agmatoptera biocellata*	ND2	Extracted	MW357302	1032
St*agmatoptera biocellata*	ND3	Extracted	MW357302	354
St*agmatoptera biocellata*	ND4	Extracted	MW357302	1338
St*agmatoptera biocellata*	ND4L	Extracted	MW357302	282
St*agmatoptera biocellata*	ND5	Extracted	MW357302	1720
St*agmatoptera biocellata*	ND6	Extracted	MW357302	501
St*agmatoptera* sp. MN117	12S	Extracted	EF383220	381
St*agmatoptera* sp. MN117	16S	Extracted	EF383380	558
St*agmatoptera* sp. MN117	ATP6	Missing	NA	0
Stag*matoptera* sp. MN117	ATP8	Missing	NA	0
Stag*matoptera* sp. MN117	COX1	Extracted	EF383863	1414
S*tagmatoptera* sp. MN117	COX2	Extracted	EF383990	621

Species	Gene	Status	Accession	Length(bp)
St*agmatoptera* sp. MN117	COX3	Missing	NA	0
Stag*matoptera* sp. MN117	CYTB	Missing	NA	0
Stag*matoptera* sp. MN117	ND1	Missing	NA	0
Stag*matoptera* sp. MN117	ND2	Missing	NA	0
Stag*matoptera* sp. MN117	ND3	Missing	NA	0
Stag*matoptera* sp. MN117	ND4	Extracted	FJ802531	1278
St*agmatoptera* sp. MN117	ND4L	Missing	NA	0
Stag*matoptera* sp. MN117	ND5	Missing	NA	0
Stag*matoptera* sp. MN117	ND6	Missing	NA	0
Stag*momantis carolina*	12S	Extracted	EF383165	378
St*agmomantis carolina*	16S	Extracted	EF383325	454
St*agmomantis carolina*	ATP6	Missing	NA	0
Stag*momantis carolina*	ATP8	Missing	NA	0
Stag*momantis carolina*	COX1	Extracted	EF383809	1414
St*agmomantis carolina*	COX2	Extracted	AY491289	563
St*agmomantis carolina*	COX3	Missing	NA	0
Stag*momantis carolina*	CYTB	Missing	NA	0
Stag*momantis carolina*	ND1	Missing	NA	0
Stag*momantis carolina*	ND2	Missing	NA	0
Stag*momantis carolina*	ND3	Missing	NA	0
Stag*momantis carolina*	ND4	Extracted	FJ802438	1278
St*agmomantis carolina*	ND4L	Missing	NA	0
Stag*momantis carolina*	ND5	Missing	NA	0
Stag*momantis carolina*	ND6	Missing	NA	0
Stag*momantis limbata*	12S	Extracted	EF383168	378
St*agmomantis limbata*	16S	Extracted	EF383328	538
St*agmomantis limbata*	ATP6	Missing	NA	0
Stag*momantis limbata*	ATP8	Missing	NA	0
Stag*momantis limbata*	COX1	Extracted	EF383812	1402
St*agmomantis limbata*	COX2	Extracted	AY491297	563
St*agmomantis limbata*	COX3	Missing	NA	0
Stag*momantis limbata*	CYTB	Missing	NA	0
Stag*momantis limbata*	ND1	Missing	NA	0
Stag*momantis limbata*	ND2	Missing	NA	0
Stag*momantis limbata*	ND3	Missing	NA	0
Stag*momantis limbata*	ND4	Extracted	FJ802446	1278
St*agmomantis limbata*	ND4L	Missing	NA	0
Stag*momantis limbata*	ND5	Missing	NA	0
Stag*momantis limbata*	ND6	Missing	NA	0
Stag*momantis* sp. MN027	12S	Extracted	FJ805982	372
St*agmomantis* sp. MN027	16S	Extracted	FJ806158	496
St*agmomantis* sp. MN027	ATP6	Missing	NA	0
Stag*momantis* sp. MN027	ATP8	Missing	NA	0

Species	Gene	Status	Accession	Length(bp)
Stag*momantis* sp. MN027	COX1	Extracted	FJ802761	1424
S*tagmomantis* sp. MN027	COX2	Extracted	AY491293	563
St*agmomantis* sp. MN027	COX3	Missing	NA	0
Stag*momantis* sp. MN027	CYTB	Missing	NA	0
Stag*momantis* sp. MN027	ND1	Missing	NA	0
Stag*momantis* sp. MN027	ND2	Missing	NA	0
Stag*momantis* sp. MN027	ND3	Missing	NA	0
Stag*momantis* sp. MN027	ND4	Extracted	FJ802442	1278
S*tagmomantis* sp. MN027	ND4L	Missing	NA	0
Stag*momantis* sp. MN027	ND5	Missing	NA	0
Stag*momantis* sp. MN027	ND6	Missing	NA	0
Stag*momantis vicina*	12S	Extracted	FJ805981	373
St*agmomantis vicina*	16S	Extracted	FJ806157	438
St*agmomantis vicina*	ATP6	Missing	NA	0
Stag*momantis vicina*	ATP8	Missing	NA	0
Stag*momantis vicina*	COX1	Extracted	FJ802760	1427
St*agmomantis vicina*	COX2	Extracted	AY491290	535
St*agmomantis vicina*	COX3	Missing	NA	0
Stag*momantis vicina*	CYTB	Missing	NA	0
Stag*momantis vicina*	ND1	Missing	NA	0
Stag*momantis vicina*	ND2	Missing	NA	0
Stag*momantis vicina*	ND3	Missing	NA	0
Stag*momantis vicina*	ND4	Extracted	FJ802439	1278
S*tagmomantis vicina*	ND4L	Missing	NA	0
Stag*momantis vicina*	ND5	Missing	NA	0
Stag*momantis vicina*	ND6	Missing	NA	0
Stat*ilia agresta*	12S	Extracted	OM202556	789
St*atilia agresta*	16S	Extracted	OM202556	1316
St*atilia agresta*	ATP6	Extracted	OM202556	681
St*atilia agresta*	ATP8	Extracted	OM202556	159
St*atilia agresta*	COX1	Extracted	OM202556	1536
St*atilia agresta*	COX2	Extracted	OM202556	685
St*atilia agresta*	COX3	Extracted	OM202556	792
St*atilia agresta*	CYTB	Extracted	OM202556	1140
St*atilia agresta*	ND1	Extracted	OM202556	936
St*atilia agresta*	ND2	Extracted	OM202556	1029
St*atilia agresta*	ND3	Extracted	OM202556	354
St*atilia agresta*	ND4	Extracted	OM202556	1338
St*atilia agresta*	ND4L	Extracted	OM202556	282
St*atilia agresta*	ND5	Extracted	OM202556	1720
St*atilia agresta*	ND6	Extracted	OM202556	504
St*atilia apicalis*	12S	Extracted	FJ805995	370
St*atilia apicalis*	16S	Extracted	FJ806171	514

Species	Gene	Status	Accession	Length(bp)
Statilia apicalis	ATP6	Missing	NA	0
Statilia apicalis	ATP8	Missing	NA	0
Statilia apicalis	COX1	Extracted	FJ802774	1427
Statilia apicalis	COX2	Extracted	AY491313	563
Statilia apicalis	COX3	Missing	NA	0
Statilia apicalis	CYTB	Missing	NA	0
Statilia apicalis	ND1	Missing	NA	0
Statilia apicalis	ND2	Missing	NA	0
Statilia apicalis	ND3	Missing	NA	0
Statilia apicalis	ND4	Extracted	FJ802463	1278
Statilia apicalis	ND4L	Missing	NA	0
Statilia apicalis	ND5	Missing	NA	0
Statilia apicalis	ND6	Missing	NA	0
Statilia flavobrunnea	12S	Extracted	OK542391	793
Statilia flavobrunnea	16S	Extracted	OK542391	1311
Statilia flavobrunnea	ATP6	Extracted	OK542391	681
Statilia flavobrunnea	ATP8	Extracted	OK542391	159
Statilia flavobrunnea	COX1	Extracted	OK542391	1536
Statilia flavobrunnea	COX2	Extracted	OK542391	685
Statilia flavobrunnea	COX3	Extracted	OK542391	789
Statilia flavobrunnea	CYTB	Extracted	OK542391	1137
Statilia flavobrunnea	ND1	Extracted	OK542391	936
Statilia flavobrunnea	ND2	Extracted	OK542391	1029
Statilia flavobrunnea	ND3	Extracted	OK542391	354
Statilia flavobrunnea	ND4	Extracted	OK542391	1338
Statilia flavobrunnea	ND4L	Extracted	OK542391	282
Statilia flavobrunnea	ND5	Extracted	OK542391	1720
Statilia flavobrunnea	ND6	Extracted	OK542391	504
Statilia maculata	12S	Extracted	KX434809	773
Statilia maculata	16S	Extracted	KX434809	1314
Statilia maculata	ATP6	Extracted	KX434809	678
Statilia maculata	ATP8	Extracted	KX434809	159
Statilia maculata	COX1	Extracted	KX434809	1536
Statilia maculata	COX2	Extracted	KX434809	685
Statilia maculata	COX3	Extracted	KX434809	792
Statilia maculata	CYTB	Extracted	KX434809	1140
Statilia maculata	ND1	Extracted	KX434809	936
Statilia maculata	ND2	Extracted	KX434809	1029
Statilia maculata	ND3	Extracted	KX434809	354
Statilia maculata	ND4	Extracted	KX434809	1338
Statilia maculata	ND4L	Extracted	KX434809	282
Statilia maculata	ND5	Extracted	KX434809	1720
Statilia maculata	ND6	Extracted	KX434809	504

Species	Gene	Status	Accession	Length(bp)
Statilia nemoralis	12S	Extracted	FJ806002	370
Statilia nemoralis	16S	Extracted	FJ806177	514
Statilia nemoralis	ATP6	Missing	NA	0
Statilia nemoralis	ATP8	Missing	NA	0
Statilia nemoralis	COX1	Extracted	FJ802781	1427
Statilia nemoralis	COX2	Extracted	FJ806891	614
Statilia nemoralis	COX3	Missing	NA	0
Statilia nemoralis	CYTB	Missing	NA	0
Statilia nemoralis	ND1	Missing	NA	0
Statilia nemoralis	ND2	Missing	NA	0
Statilia nemoralis	ND3	Missing	NA	0
Statilia nemoralis	ND4	Extracted	FJ802493	1278
Statilia nemoralis	ND4L	Missing	NA	0
Statilia nemoralis	ND5	Missing	NA	0
Statilia nemoralis	ND6	Missing	NA	0
Statilia sp. MN255	12S	Extracted	FJ806070	305
Statilia sp. MN255	16S	Extracted	FJ806246	514
Statilia sp. MN255	ATP6	Missing	NA	0
Statilia sp. MN255	ATP8	Missing	NA	0
Statilia sp. MN255	COX1	Extracted	FJ802849	1427
Statilia sp. MN255	COX2	Extracted	FJ806960	670
Statilia sp. MN255	COX3	Missing	NA	0
Statilia sp. MN255	CYTB	Missing	NA	0
Statilia sp. MN255	ND1	Missing	NA	0
Statilia sp. MN255	ND2	Missing	NA	0
Statilia sp. MN255	ND3	Missing	NA	0
Statilia sp. MN255	ND4	Extracted	FJ802653	1278
Statilia sp. MN255	ND4L	Missing	NA	0
Statilia sp. MN255	ND5	Missing	NA	0
Statilia sp. MN255	ND6	Missing	NA	0
Tamolanica tamolana	12S	Extracted	NC 007702	777
Tamolanica tamolana	16S	Extracted	NC 007702	1318
Tamolanica tamolana	ATP6	Extracted	NC 007702	678
Tamolanica tamolana	ATP8	Extracted	NC 007702	159
Tamolanica tamolana	COX1	Extracted	NC 007702	1539
Tamolanica tamolana	COX2	Extracted	NC 007702	685
Tamolanica tamolana	COX3	Extracted	NC 007702	792
Tamolanica tamolana	CYTB	Extracted	NC 007702	1136
Tamolanica tamolana	ND1	Extracted	NC 007702	936
Tamolanica tamolana	ND2	Extracted	NC 007702	1028
Tamolanica tamolana	ND3	Extracted	NC 007702	354
Tamolanica tamolana	ND4	Extracted	NC 007702	1338
Tamolanica tamolana	ND4L	Extracted	NC 007702	282

Species	Gene	Status	Accession	Length(bp)
Tamolanica tamolana	ND5	Extracted	NC 007702	1720
Tamolanica tamolana	ND6	Extracted	NC 007702	504
Tarachomantis alaotrana	12S	Extracted	KX434862	780
Tarachomantis alaotrana	16S	Extracted	KX434862	1316
Tarachomantis alaotrana	ATP6	Extracted	KX434862	678
Tarachomantis alaotrana	ATP8	Extracted	KX434862	159
Tarachomantis alaotrana	COX1	Extracted	KX434862	1539
Tarachomantis alaotrana	COX2	Extracted	KX434862	685
Tarachomantis alaotrana	COX3	Extracted	KX434862	789
Tarachomantis alaotrana	CYTB	Extracted	KX434862	1137
Tarachomantis alaotrana	ND1	Extracted	KX434862	936
Tarachomantis alaotrana	ND2	Extracted	KX434862	1029
Tarachomantis alaotrana	ND3	Extracted	KX434862	354
Tarachomantis alaotrana	ND4	Extracted	KX434862	1338
Tarachomantis alaotrana	ND4L	Extracted	KX434862	282
Tarachomantis alaotrana	ND5	Extracted	KX434862	1723
Tarachomantis alaotrana	ND6	Extracted	KX434862	504
Tarachomantis caldwellii	12S	Missing	NA	0
Tarachomantis caldwellii	16S	Extracted	FJ806281	513
Tarachomantis caldwellii	ATP6	Missing	NA	0
Tarachomantis caldwellii	ATP8	Missing	NA	0
Tarachomantis caldwellii	COX1	Extracted	FJ802880	644
Tarachomantis caldwellii	COX2	Extracted	FJ806998	670
Tarachomantis caldwellii	COX3	Missing	NA	0
Tarachomantis caldwellii	CYTB	Missing	NA	0
Tarachomantis caldwellii	ND1	Missing	NA	0
Tarachomantis caldwellii	ND2	Missing	NA	0
Tarachomantis caldwellii	ND3	Missing	NA	0
Tarachomantis caldwellii	ND4	Extracted	FJ802704	1278
Tarachomantis caldwellii	ND4L	Missing	NA	0
Tarachomantis caldwellii	ND5	Missing	NA	0
Tarachomantis caldwellii	ND6	Missing	NA	0
Tenodera angustipennis	12S	Extracted	KX434852	797
Tenodera angustipennis	16S	Extracted	KX434852	1312
Tenodera angustipennis	ATP6	Extracted	KX434852	678
Tenodera angustipennis	ATP8	Extracted	KX434852	159
Tenodera angustipennis	COX1	Extracted	KX434852	1542
Tenodera angustipennis	COX2	Extracted	KX434852	685
Tenodera angustipennis	COX3	Extracted	KX434852	792
Tenodera angustipennis	CYTB	Extracted	KX434852	1137
Tenodera angustipennis	ND1	Extracted	KX434852	936
Tenodera angustipennis	ND2	Extracted	KX434852	1029
Tenodera angustipennis	ND3	Extracted	KX434852	354

Species	Gene	Status	Accession	Length(bp)
Tenodera angustipennis	ND4	Extracted	KX434852	1336
Tenodera angustipennis	ND4L	Extracted	KX434852	282
Tenodera angustipennis	ND5	Extracted	KX434852	1723
Tenodera angustipennis	ND6	Extracted	KX434852	504
Tenodera aridifolia brevicollis	12S	Extracted	OK542393	818
Tenodera aridifolia brevicollis	16S	Extracted	OK542393	1313
Tenodera aridifolia brevicollis	ATP6	Extracted	OK542393	681
Tenodera aridifolia brevicollis	ATP8	Extracted	OK542393	159
Tenodera aridifolia brevicollis	COX1	Extracted	OK542393	1551
Tenodera aridifolia brevicollis	COX2	Extracted	OK542393	685
Tenodera aridifolia brevicollis	COX3	Extracted	OK542393	789
Tenodera aridifolia brevicollis	CYTB	Extracted	OK542393	1137
Tenodera aridifolia brevicollis	ND1	Extracted	OK542393	936
Tenodera aridifolia brevicollis	ND2	Extracted	OK542393	1029
Tenodera aridifolia brevicollis	ND3	Extracted	OK542393	354
Tenodera aridifolia brevicollis	ND4	Extracted	OK542393	1338
Tenodera aridifolia brevicollis	ND4L	Extracted	OK542393	282
Tenodera aridifolia brevicollis	ND5	Extracted	OK542393	1724
Tenodera aridifolia brevicollis	ND6	Extracted	OK542393	504
Tenodera costalis	12S	Extracted	EF383166	377
Tenodera costalis	16S	Extracted	EF383326	548
Tenodera costalis	ATP6	Missing	NA	0
Tenodera costalis	ATP8	Missing	NA	0
Tenodera costalis	COX1	Extracted	EF383810	1413
Tenodera costalis	COX2	Extracted	AY491291	535
Tenodera costalis	COX3	Missing	NA	0
Tenodera costalis	CYTB	Missing	NA	0
Tenodera costalis	ND1	Missing	NA	0
Tenodera costalis	ND2	Missing	NA	0
Tenodera costalis	ND3	Missing	NA	0
Tenodera costalis	ND4	Extracted	FJ802440	1278
Tenodera costalis	ND4L	Missing	NA	0
Tenodera costalis	ND5	Missing	NA	0
Tenodera costalis	ND6	Missing	NA	0
Tenodera sinensis	12S	Extracted	KX673199	812
Tenodera sinensis	16S	Extracted	KX673199	1311
Tenodera sinensis	ATP6	Extracted	KX673199	681
Tenodera sinensis	ATP8	Extracted	KX673199	159
Tenodera sinensis	COX1	Extracted	KX673199	1539
Tenodera sinensis	COX2	Extracted	KX673199	685
Tenodera sinensis	COX3	Extracted	KX673199	792
Tenodera sinensis	CYTB	Extracted	KX673199	1137
Tenodera sinensis	ND1	Extracted	KX673199	936

Species	Gene	Status	Accession	Length(bp)
Tenodera sinensis	ND2	Extracted	KX673199	1029
Tenodera sinensis	ND3	Extracted	KX673199	354
Tenodera sinensis	ND4	Extracted	KX673199	1338
Tenodera sinensis	ND4L	Extracted	KX673199	282
Tenodera sinensis	ND5	Extracted	KX673199	1720
Tenodera sinensis	ND6	Extracted	KX673199	504
Titanodula formosana	12S	Extracted	KR703238	803
Titanodula formosana	16S	Extracted	KR703238	1311
Titanodula formosana	ATP6	Extracted	KR703238	678
Titanodula formosana	ATP8	Extracted	KR703238	159
Titanodula formosana	COX1	Extracted	KR703238	1542
Titanodula formosana	COX2	Extracted	KR703238	685
Titanodula formosana	COX3	Extracted	KR703238	792
Titanodula formosana	CYTB	Extracted	KR703238	1136
Titanodula formosana	ND1	Extracted	KR703238	936
Titanodula formosana	ND2	Extracted	KR703238	1028
Titanodula formosana	ND3	Extracted	KR703238	354
Titanodula formosana	ND4	Extracted	KR703238	1338
Titanodula formosana	ND4L	Extracted	KR703238	282
Titanodula formosana	ND5	Extracted	KR703238	1720
Titanodula formosana	ND6	Extracted	KR703238	501
Vates pectinacornis	12S	Extracted	EF383159	379
Vates pectinacornis	16S	Extracted	EF383319	522
Vates pectinacornis	ATP6	Missing	NA	0
Vates pectinacornis	ATP8	Missing	NA	0
Vates pectinacornis	COX1	Extracted	EF383803	1414
Vates pectinacornis	COX2	Extracted	AY491280	563
Vates pectinacornis	COX3	Missing	NA	0
Vates pectinacornis	CYTB	Missing	NA	0
Vates pectinacornis	ND1	Missing	NA	0
Vates pectinacornis	ND2	Missing	NA	0
Vates pectinacornis	ND3	Missing	NA	0
Vates pectinacornis	ND4	Extracted	FJ802429	1278
Vates pectinacornis	ND4L	Missing	NA	0
Vates pectinacornis	ND5	Missing	NA	0
Vates pectinacornis	ND6	Missing	NA	0
Vates sp. MN118	12S	Extracted	FJ806019	373
Vates sp. MN118	16S	Extracted	FJ806194	514
Vates sp. MN118	ATP6	Missing	NA	0
Vates sp. MN118	ATP8	Missing	NA	0
Vates sp. MN118	COX1	Extracted	FJ802798	1427
Vates sp. MN118	COX2	Extracted	FJ806908	670
Vates sp. MN118	COX3	Missing	NA	0

Species	Gene	Status	Accession	Length(bp)
Vate*s* sp. MN118	CYTB	Missing	NA	0
Vate*s* sp. MN118	ND1	Missing	NA	0
Vate*s* sp. MN118	ND2	Missing	NA	0
Vate*s* sp. MN118	ND3	Missing	NA	0
Vate*s* sp. MN118	ND4	Extracted	FJ802532	1278
V*ates* sp. MN118	ND4L	Missing	NA	0
Vate*s* sp. MN118	ND5	Missing	NA	0
Vate*s* sp. MN118	ND6	Missing	NA	0
Xyst*ropeltis* sp. MN268	12S	Extracted	EF383299	375
Xy*stropeltis* sp. MN268	16S	Extracted	EF383459	389
Xy*stropeltis* sp. MN268	ATP6	Missing	NA	0
Xyst*ropeltis* sp. MN268	ATP8	Missing	NA	0
Xyst*ropeltis* sp. MN268	COX1	Missing	NA	0
Xyst*ropeltis* sp. MN268	COX2	Extracted	EF384070	351
Xy*stropeltis* sp. MN268	COX3	Missing	NA	0
Xyst*ropeltis* sp. MN268	CYTB	Missing	NA	0
Xyst*ropeltis* sp. MN268	ND1	Missing	NA	0
Xyst*ropeltis* sp. MN268	ND2	Missing	NA	0
Xyst*ropeltis* sp. MN268	ND3	Missing	NA	0
Xyst*ropeltis* sp. MN268	ND4	Extracted	FJ802664	389
Xy*stropeltis* sp. MN268	ND4L	Missing	NA	0
Xyst*ropeltis* sp. MN268	ND5	Missing	NA	0
Xyst*ropeltis* sp. MN268	ND6	Missing	NA	0
