# Supplementary material for: A Mitogenome-Based Phylogenetic Framework for Mantidae (Mantodea: Mantoidea): Systematic Implications and Spatio-Temporal Dynamics
Source: Insects. 2026 Aug 10;17(8):830. doi: 10.3390/insects17080830 (PMC13513240; doi:10.3390/insects17080830)
Supplement: Supplementary file 1 [file insects-17-00830-s001.zip › Table S4.pdf]

Table S4 Ancestral range estimation for dated phylogeny of Mantidae (A: Palearctic, B: Nearctic, C: Neotropical, D: Afrotropical, E: PIP, F: Australasia ,G:SE Asia, H: Madagascar).

Species	Region	Partition	Source
Antemna rapax	Costa Rica	C	Svenson & Whiting(2009)
Archimantis latistyla	Australia	F	This study
Archimantis quinquelobata	Australia	F	This study
Archimantis sobrina	Australia	F	This study
Asiadodis yunnanensis	Yunnan, China	G	Xu, et al(2021)
Austromantis albomarginata	Australia	F	This study
Austrovates variegata	Australia	F	This study
Camelomantis moultoni	Borneo	G	Svenson & Whiting(2009)
Cataspilota guineensis	Ghana	D	Svenson & Whiting(2009)
Choeradodis rhombicollis	Panama	C	Svenson & Whiting(2009)
Choeradodis stalii	Peru	C	Svenson & Whiting(2009)
Hierodula chinensis	Hunan,China	A	Liu, et al(2022)
Hierodula latipennis	SE Asia	G	inaturalist
Hierodula longa	Yunnan, China	G	Wang et al(2022)
Hierodula maculata	Guangxi,China	G	Wang et al(2022)
Hierodula majuscula	SE Asia, Australia	FG	inaturalist
Hierodula membranacea	PIP	E	inaturalist
Hierodula multispina	Yunnan, China	G	inaturalist
Hierodula patellifera	Palearctic, Nearctic, PIP,Australia	ABEG	inaturalist
Hierodula pulchripes	SE Asia	G	This study
Hierodula schultzei	PNG	F	Svenson & Whiting(2009)
Hierodula sp.	SE Asia	G	This study
Hierodula timorensis	SE Asia	G	This study
Hierodula zhangi	Yunnan, China	G	Wang et al(2022)
Ephierodula celebensis	Borneo	G	Svenson & Whiting(2009)
Ephierodula reticulata	Borneo	G	Svenson & Whiting(2009)
Mantis religiosa	Hequan, Dingbian, Shannx	A	Zhang et al(2017)
Mesopteryx alata	Taiwan, China	G	Wang et al(2022)
Omomantis sp.	captive bred	D	Svenson & Whiting(2009)
Orthodera ministralis	Australian	F	Wang et al(2022)
Orthodera novaezealandiae	Australian	F	Svenson & Whiting(2009)
Orthodera sp.	Australia	F	Svenson & Whiting(2009)
Oxyopsis peruviana	Neotropical	C	This study
Oxyopsis sp.	Bolivia	C	Svenson & Whiting(2009)
Parastagmatoptera sp.	Panama, Costa Rica	C	Svenson & Whiting(2009)
Phasmomantis sp.	Neotropical	C	This study

Species	Region	Partition	Source
Plistospilota sp.	Ethiopian realm	D	inaturalist
Pnigomantis medioconstricta	Oriental realm	EF	Wang et al(2022)
Polyspilota aeruginosa	Ghana	D	Svenson & Whiting(2009)
Prohierodula ornatipennis	Ghana	D	Svenson & Whiting(2009)
Prohierodula picta	Ethiopian	D	Wang et al(2022)
Pseudomantis albofimbriata	Australia	F	This study
Pseudovates chlorophaea	Nearctic realm	B	Wang et al(2022)
Pseudovates cingulata	Peru	C	Svenson & Whiting(2009)
Pseudovates denticulata	French Guiana	C	Svenson & Whiting(2009)
Pseudovates peruviana	Neotropical	C	inaturalist
Rhombodera basalis	SE Asia	G	This study
Rhombodera brachynota	Mengla, Yunnan	G	Zhang et al(2017)
Rhombodera stalii	Asia, Australia, Borneo	AFG	Svenson & Whiting(2009)
Rhombodera valida	Mengla, Yunnan	G	Zhang et al(2017)
Sphodromantis lineola	Africa	D	Zhang et al(2018)
Sphodromantis viridis	Palearctic, Nearctic, Afrotropical	ABD	This study
Sphodropoda sp.	Australia	F	Svenson & Whiting(2009)
Sphodropoda sp.	Australia	F	inaturalist
Stagmatoptera biocellata	Neotropical	C	inaturalist
Stagmatoptera sp.	Peru	C	Svenson & Whiting(2009)
Stagmomantis carolina	United States	B	Svenson & Whiting(2009)
Stagmomantis limbata	United States	B	Svenson & Whiting(2009)
Stagmomantis sp.	Panama, Costa Rica, Nicaragua	C	Svenson & Whiting(2009)
Stagmomantis vicina	Costa Rica	C	Svenson & Whiting(2009)
Statilia agresta	China	AG	inaturalist
Statilia apicalis	Australia	F	Svenson & Whiting(2009)
Statilia flavobrunnea	Guangdong,China	G	Wang et al(2022)
Statilia maculata	Zhejiang,China	A	Zhang et al(2019)
Statilia nemoralis	India	E	Svenson & Whiting(2009)
Statilia sp.	India	E	Svenson & Whiting(2009)
Tamolanica tamolana	Herowana Crater	F	inaturalist
Tarachomantis alaotrana	Madagascar	H	inaturalist
Tarachomantis caldwellii	Madagascar	H	Svenson & Whiting(2009)
Tenodera angustipennis	Tongren, Guizhou	A	Wang et al(2022)
Tenodera aridifolia brevicollis	Yunnan, China	G	Wang et al(2022)
Tenodera costalis	Australia	F	Svenson & Whiting(2009)
Titanodula formosana	Taiwan, China	G	Tian et al.(2017)
Vates pectinacornis	Panama	C	Svenson & Whiting(2009)

Species	Region	Partition	Source
Vates sp.	Peru	C	Svenson & Whiting(2009)
Tenodera sinensis	Palearctic, Nearctic, Neotropical, PIP, SE Asia	ABCEG	inaturalist
Xystropeltis sp.	Mexico	C	Svenson & Whiting(2009)
