# Supplementary material for: A Mitogenome-Based Phylogenetic Framework for Mantidae (Mantodea: Mantoidea): Systematic Implications and Spatio-Temporal Dynamics
Source: Insects. 2026 Aug 10;17(8):830. doi: 10.3390/insects17080830 (PMC13513240; doi:10.3390/insects17080830)
Supplement: Supplementary file 1 [file insects-17-00830-s001.zip › Table S5.pdf]

Table S5. Scores for BioGeoBEARS models.

Model	LnL	numparams	d	e	j	AICc	AICc_wt
DEC	-146.4	2	0.26	5	0	297	0.64
DEC+J	-145.9	3	0.24	4.88	0.0051	298.2	0.36
DIVALIKE	-163	2	0.17	0.15	0	330.3	3.90E-08
DIVALIKE+J	-154.4	3	0.11	0.45	0.017	315.2	7.20E-05
BAYAREALIKE	-193	2	0.095	0.84	0	390.1	3.90E-21
BAYAREALIKE+J	-153.2	3	0.12	1.91	0.016	312.7	0.0003
