# Supplementary material for: A Mitogenome-Based Phylogenetic Framework for Mantidae (Mantodea: Mantoidea): Systematic Implications and Spatio-Temporal Dynamics
Source: Insects. 2026 Aug 10;17(8):830. doi: 10.3390/insects17080830 (PMC13513240; doi:10.3390/insects17080830)
Supplement: Supplementary file 1 [file insects-17-00830-s001.zip › Table S6.pdf]

Table S6. Primers used to verify gene rearrangement.

species	Primer Name	Sequence (5'–3')
Rhombodera basalis	TL-J-7	ATGCCTGATTCAAAAGGGTT
	TL-N-1247	TATTGGCTTGAAAGAGGAGG
Oxyopsis peruviana	TI-J-46	AAGCCCTTTCCAATCACAA
	TL-N-688	TGGGGTATGAACCCAATAG
Hierodula timorensis	TL-J-138	CTCCTGAATAGGAGCATGAA
	TL-N-1675	ATTCTTGCTGATAAGGGTGG
Orthodera novaezealandiae	TL-J-5892	ATGAATGAAATCAAGGGTCA
	TL-N-6532	AATAAAATTAGGAGGAATTAATCA
Euchomenella sp.	TL-J-5754	TCACGACTACCTTTTTCATTACGA
	TL-N-6446	AGCAGCTTTTATTTGATCAACACA
Phasmomantella sp.	TL-J-5978	TCATGAATGAAATCAAGGTGCTCT
	TL-N-6537	AGTATAACATTGAAGCTGTTATTGAGA
