# Supplementary material for: A Mitogenome-Based Phylogenetic Framework for Mantidae (Mantodea: Mantoidea): Systematic Implications and Spatio-Temporal Dynamics
Source: Insects. 2026 Aug 10;17(8):830. doi: 10.3390/insects17080830 (PMC13513240; doi:10.3390/insects17080830)
Supplement: Supplementary file 1 [file insects-17-00830-s001.zip › Figure S1.pdf]

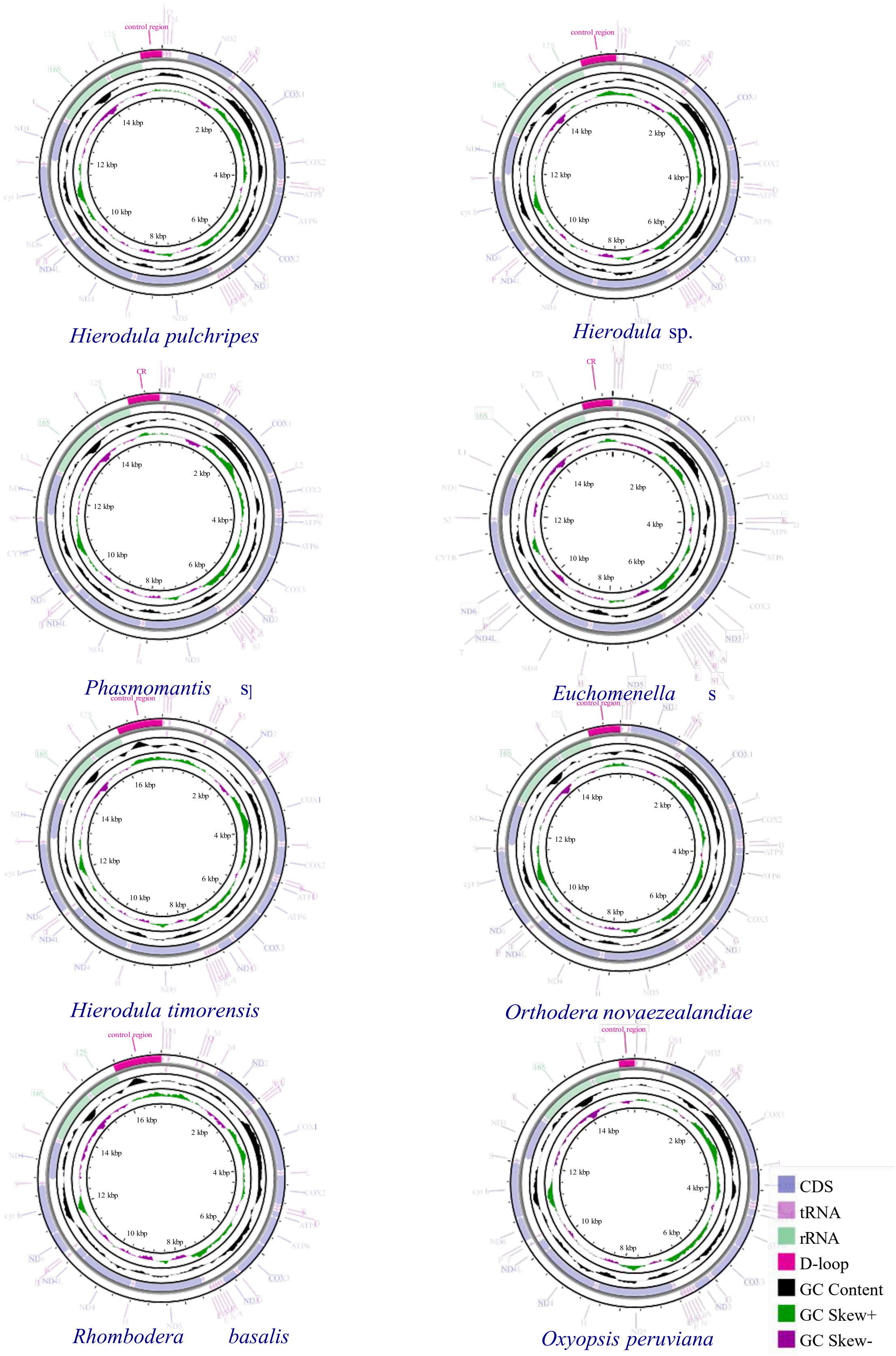

Figure S1. Circular maps of the 16 mitogenomes. Genes in the outer circle are encoded on the heavy strand, whereas those in the inner circle are encoded on the light strand.
